# Serotype-specific differences in short- and longer-term mortality following invasive pneumococcal disease

**DOI:** 10.1017/S0950268816000856

**Published:** 2016-05-19

**Authors:** G. J. HUGHES, L. B. WRIGHT, K. E. CHAPMAN, D. WILSON, R. GORTON

**Affiliations:** 1 Academic Unit of Public Health, University of Leeds, Leeds, UK; 2 Public Health England North East, Citygate, Gallowgate, Newcastle upon Tyne, UK

**Keywords:** Mortality, pneumococcal infections, serotype, survival analysis

## Abstract

Invasive pneumococcal disease (IPD), caused by infection with *Streptococcus pneumoniae*, has a substantial global burden. There are over 90 known serotypes of *S. pneumoniae* with a considerable body of evidence supporting serotype-specific mortality rates immediately following IPD. This is the first study to consider the association between serotype and longer-term mortality following IPD. Using enhanced surveillance data from the North East of England we assessed both the short-term (30-day) and longer-term (⩽7 years) independent adjusted associations between individual serotypes and mortality following IPD diagnosis using logistic regression and extended Cox proportional hazards models. Of the 1316 cases included in the analysis, 243 [18·5%, 95% confidence interval (CI) 16·4–20·7] died within 30 days of diagnosis. Four serotypes (3, 6A, 9N, 19 F) were significantly associated with overall increased 30-day mortality. Effects were observable only for older adults (⩾60 years). After extension of the window to 12 months and 36 months, one serotype was associated with significantly increased mortality at 12 months (19 F), but no individual serotypes were associated with increased mortality at 36 months. Two serotypes had statistically significant hazard ratios (HR) for longer-term mortality: serotype 1 for reduced mortality (HR 0·51, 95% CI 0·30–0·86) and serotype 9N for increased mortality (HR 2·30, 95% CI 1·29–4·37). The association with serotype 9N was no longer observed after limiting survival analysis to an observation period starting 30 days after diagnosis. This study supports the evidence for associations between serotype and short-term (30-day) mortality following IPD and provides the first evidence for the existence of statistically significant associations between individual serotypes and longer-term variation in mortality following IPD.

## INTRODUCTION

Invasive pneumococcal disease (IPD), caused by infection with *Streptococcus pneumoniae*, has a substantial global burden in young children [1] and adults [2]. There are over 90 known serotypes of *S. pneumoniae*, differentiation of which is based on the composition of the polysaccharide capsule [3]. Different serotypes induce different immune responses and, together with other bacterial virulence factors and host risk factors, contribute to pathogenicity and severity of infection [3]. Given that vaccines against infection with *S. pneumoniae* are limited to certain subsets of serotypes, consideration of differential pathogenicity (and the resultant severity of infection and associated mortality) is important for maximizing the societal benefits of a vaccination programme [4].

There is a growing body of evidence to support the existence of serotype-specific mortality rates immediately following IPD [4–9]. Studies which include infection of children are limited in statistical power due to low mortality rates for this age group [4, 5], but there is clear evidence for adults that certain serotypes are associated with a relatively increased 30-day mortality compared to other serotypes [8]. In the longer-term, reduced life expectancy following pneumococcal pneumonia (⩽10 years) [10] and meningitis (⩽20 years) [11] has also been observed. Generally, septicaemia is associated with increased rates of long-term mortality (⩽6 years), although it is not clear whether this is an independent consequence of pathology, or due to comorbidities or sequelae [12]. Whatever the mechanism, such an effect is likely for pneumococcal septicaemia.

Given the likely impact of IPD on longer-term mortality, it is important to understand whether serotype-specific associations with longer-term mortality exist. Such information may help to inform cost-effectiveness studies for future vaccine formulations. Using enhanced surveillance data from the North East of England linked to registered deaths, we assessed both the short-term (30-day) and longer-term (⩽7 years) associations between individual serotypes of *S. pneumoniae* and mortality following diagnosis.

## METHODS

### Study population

The North East of England has a population of about 2·6 million persons [13], and has the least favourable self-reported general health in the country [14]. Life expectancy is among the lowest in the UK; 1·0 and 1·1 years below the national average for males and females, respectively [13]. On a small area level, the North East has a disproportionate level of social deprivation compared to other regions of the UK: 33% of small area populations of 1500 persons are within the most deprived national quintile [15].

### Surveillance data and linkage to mortality data

Cases of IPD with a specimen date between 1 April 2006 and 31 March 2013 were obtained from the North East IPD enhanced surveillance system. Full details of the surveillance system are described elsewhere [16]. Briefly, all laboratories in the region notified local health protection staff of laboratory-confirmed cases of IPD. Following notification, telephone interviews with laboratory staff and primary-/secondary-care clinicians were conducted to obtain details of risk factors (indicators for pneumococcal vaccination agreed for England plus alcohol misuse) and vaccination status. Isolates were typed at the national reference laboratory. Cases were residents of the North East of England with *S. pneumoniae* detected from a normally sterile site. Age, sex, pneumococcal vaccination status, risk factors for IPD (alcohol misuse, chronic heart disease, chronic liver disease, chronic lung disease, chronic renal disease, diabetes, immunosuppression), and clinical presentation (bacteraemic pneumonia, meningitis, septicaemia) were routinely collected for each case. Other clinical presentations found at low frequency within the dataset (e.g. endocarditis, peritonitis) were grouped into a single category. Cases were attributed to quintiles of social deprivation as described elsewhere [17]. The social deprivation index used incorporates multiple domains (income, employment, health, education, housing, crime) to characterize small area living environments. All cases where the serotype was unknown were excluded. Serotypes which each accounted for >1% of the dataset were considered individually. We limited analysis to cases without missing data for any variables and those which could be linked to mortality data. For cases where the positive specimen was taken on or after the date of death, specimen dates were adjusted to 1 day before the date of death. Cases were linked to Office for National Statistics registered deaths up to 30 September 2013 (data obtained March 2014) using unique National Health Service (NHS) numbers. Data was used in accordance with a data access agreement with Public Health England. The study was neither powered nor designed to test a specific hypothesis.

### Associations with mortality at 30 days, 12 months and 36 months post-diagnosis

A binary logistic regression model was used to assess serotype-specific differences in all-cause mortality at three time points following diagnosis with IPD (30 days, 12 months, 36 months). Only cases with follow-up to the time point were included in that analysis. For each model, variables with a single variable association with outcome of *P* < 0·2 (*χ*^2^ for variables with two categories, Wald test for those with >2) were considered for inclusion in the multivariable model; age group, sex and serotype were retained irrespective of statistical significance. A backward selection model-building strategy was used, starting with a model containing all selected variables from the single variable analysis. Variables were then tested for exclusion if their coefficient in the model had an associated *P* value >0·05 (starting with the variable with the highest *P* value). Each reduced model was evaluated for fit using a likelihood ratio test. Goodness of fit for the final model (where all parameters had an associated *P* value <0·05) was assessed using the Hosmer–Lemeshow goodness-of-fit test (with ten groups) [18] and a case classification table (cut-off 0·5). Marginal predicted probabilities of death at 30 days were obtained from the final multivariable model for each age group.

### Survival analysis

A Cox proportional hazards (PH) model was used to assess serotype-specific differences in all-cause mortality following diagnosis with IPD (defined as specimen date of the first positive test). An event was the day of death, truncated to 30 September 2013. To ensure acceptable precision at the end of the observation period, the period was limited to 7 years (truncating a small number of cases with data beyond this time point). Crude associations with mortality were assessed using the log rank test and any variables with an association of *P* < 0·2 were considered for inclusion in the Cox PH model. A backward selection model was used as for the logistic regression model. The assumption of PH of the final model was evaluated using a test of correlation of scaled Schoenfeld residuals with time. Where the PH assumption was not met (*P* < 0·05), an extended Cox PH model was specified to include time-dependent effects (as a function of the natural logarithm of time) for each variable. The improvement of the extended model to the main-effects model was assessed using a likelihood ratio test. Adjusted Kaplan–Meier survival curves were used to assess serotype-specific effects after adjusting for all variables included in the Cox PH model. We repeated the Cox PH model using an observation period starting 30 days after diagnosis.

### Statistical software

All analysis was performed using Stata v. 13.1 (StataCorp., USA).

## RESULTS

### Surveillance data and linkage to mortality data

From the full surveillance dataset, 1779/1785 (99·7%) IPD cases had NHS numbers available. Of these cases, 210 (11·8%) had no serotype data available. A further 253 cases (16·1%) had data missing for at least one variable (postcode, *n* = 26; pneumococcal vaccination status, *n* = 84; clinical presentation, *n* = 22; ⩾1 risk factor, *n* = 162) and were excluded; leaving 1316 cases for analysis (74·0% of the original dataset, Table 1). Of this dataset, 24 serotypes each accounted for >1% of cases (⩾16 cases per serotype) and were included as individual categories (Table 2). Forty-seven cases from this dataset were diagnosed with IPD on the date of death or at post-mortem; to enable inclusion of these cases in survival analysis, the specimen date was adjusted to the day before the date of death.

**Table 1.** Characteristics of IPD cases

Variable	Category*	No. of cases (%)		
		In category	Yes	No
Age group, years	0–19	195 (14·8)	—	—
	20–39	137 (10·4)	—	—
	40–59	275 (20·9)	—	—
	60–79	446 (33·9)	—	—
	⩾80	263 (20·0)		
Sex	Male	686 (52·1)	—	—
	Female	630 (47·9)	—	—
Year of diagnosis	2006–2007	209 (15·9)	—	—
	2007–2008	235 (17·9)	—	—
	2008–2009	188 (14·3)	—	—
	2009–2010	208 (15·8)	—	—
	2010–2011	203 (15·4)	—	—
	2011–2012	150 (11·4)	—	—
	2012–2013	123 (9·4)	—	—
Deprivation quintile†	Quintile 1	186 (14·1)	—	—
	Quintile 2	241 (18·3)	—	—
	Quintile 3	261 (19·8)	—	—
	Quintile 4	284 (21·6)	—	—
	Quintile 5	344 (26·1)	—	—
Pneumococcal immunization	Vaccinated	—	604 (45·9)	712 (54·1)
Clinical presentation	Meningitis	125 (9·5)	—	—
	Pneumonia	908 (69·0)	—	—
	Septicaemia	125 (9·5)	—	—
	Other	158 (12·0)	—	—
Alcohol misuse	—	—	113 (8·6)	1203 (91·4)
Chronic heart disease	—	—	291 (22·1)	1025 (77·9)
Chronic liver disease	—	—	58 (4·4)	1258 (95·6)
Chronic lung disease	—	—	323 (24·5)	993 (75·5)
Chronic renal disease	—	—	141 (10·7)	1175 (89·3)
Diabetes	—	—	151 (11·5)	1165 (88·5)
Immunosuppression	—	—	145 (11·0)	1171 (89·0)
Number of IPD risk factors‡	0	610 (46·4)	—	—
	1	396 (30·1)	—	—
	⩾2	310 (23·6)	—	—

IPD, Invasive pneumococcal disease.

* Missing category only presented where ⩾1 case had missing data for that variable.

† Quintile 1, least deprived; quintile 5, most deprived.

‡ Includes alcohol misuse, chronic heart disease, chronic liver disease, chronic lung disease, chronic renal disease, diabetes, immunosuppression.

**Table 2.** Distribution of serotypes of invasive pneumococcal disease cases

Serotype*	No. of cases	%
1	165	12·54
3	114	8·66
4	35	2·66
6A	34	2·58
6B	26	1·98
6C	33	2·51
7F	150	11·40
8	98	7·45
9N	22	1·67
9V	31	2·36
10A	16	1·22
11A	22	1·67
12F	38	2·89
14	41	3·12
18C	24	1·82
19A	117	8·89
19F	24	1·82
20	16	1·22
22F	90	6·84
23A	21	1·60
23F	35	2·66
31	16	1·22
33F	34	2·58
35F	16	1·22
Other serotypes†	98	7·45

* Serotypes which each account for >1% of the dataset.

† Serotypes 2, 5, 7, 9A, 10F, 12B, 15A, 15B, 15C, 16, 16A, 16F, 17F, 19, 21, 23, 23B, 24F, 25F, 28A, 29, 33A, 34, 35B, 38.

### Mortality at 30 days, 12 months and 36 months post-diagnosis

**Fig. 1.**
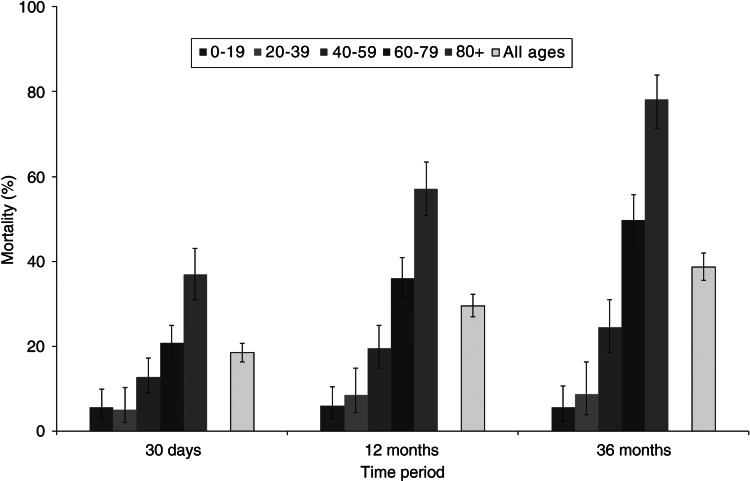
Crude mortality rates at 30 days, 12 months and 36 months post-diagnosis with invasive pneumococcal disease by age groups.

Of the 1316 cases included in the analysis, a total of 243 [18·5%, 95% confidence interval (CI) 16·4–20·7] died within 30 days of diagnosis. As expected, mortality rates were higher when the follow-up period was extended to 12 months (364 deaths from the 1233 cases with sufficient follow-up time; 29·5%, 95% CI 27·0–32·2) and 36 months (352 deaths from the 911 cases with sufficient follow-up time; 38·6%, 95% CI 35·4–41·9). A similar pattern of increasing mortality rates with increasing age groups was seen for all three time points (Fig. 1).

**Fig. 2.**
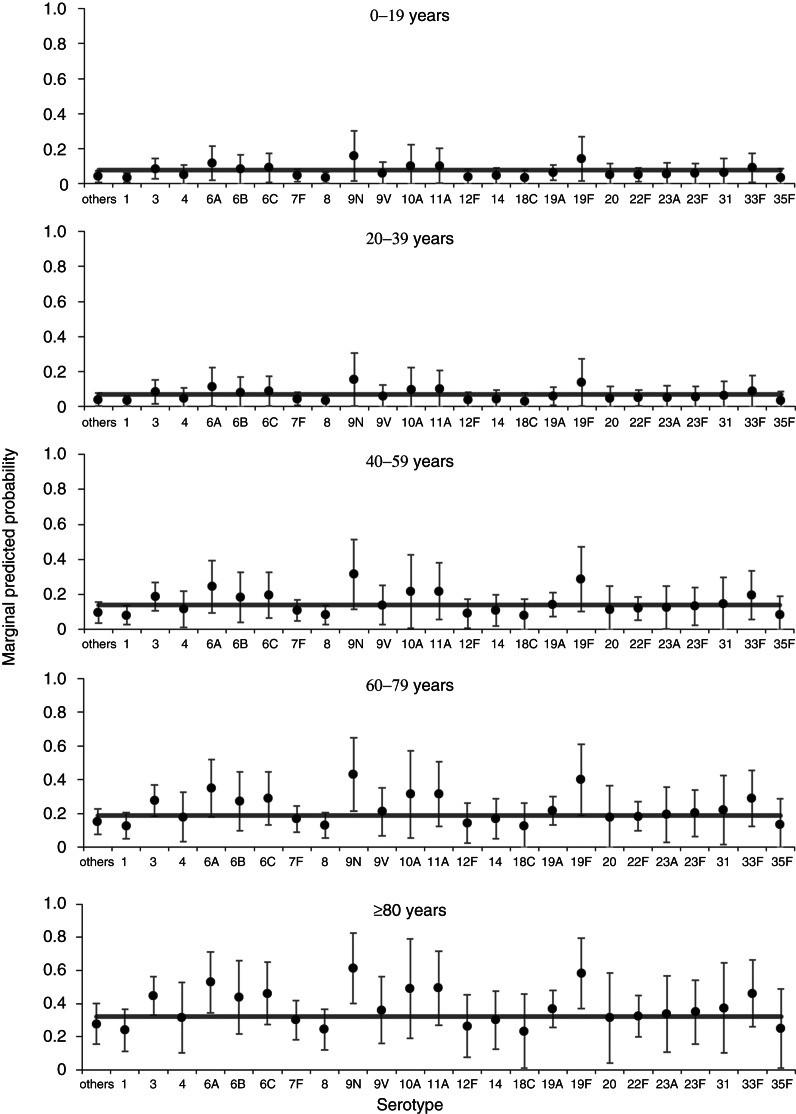
Predicted marginal probabilities of survival after 30 days following diagnosis with invasive pneumococcal disease by serotype. The grey line indicates the average for each age group. Probabilities are adjusted for sex, deprivation, serotype, clinical presentation and number of risk factors.

A multivariable model for associations with mortality at 30 days contained age group, sex, serotype (retained irrespective of significance) plus deprivation, clinical presentation and number of risk factors (Supplementary Table S1, Table 3). The model has an overall acceptable goodness of fit (*χ*^2^ = 8·46, d.f. = 8, *P* = 0·390), no reduction in fit compared to the starting model (*χ*^2^ = 6·30, d.f. = 7, *P* = 0·505) and correct classification of 82% of cases. From this model, four serotypes (3, 6A, 9N, 19 F) were significantly associated with an overall increased mortality compared to the group of other serotypes. There is little variation in predicted probabilities of mortality at 30 days by serotype in children and younger adults (Fig. 2). Those serotypes found to have an overall significant association with increased mortality at 30 days are only predicted to have a rate significantly above the age group average for older adults (⩾60 years).

**Table 3.** Crude and adjusted associations with mortality at 30 days, 12 months and 36 months post-diagnosis with invasive pneumococcal disease

Variable	Category	30-day survival				12-month survival				36-month survival			
		Single variable analysis		Multivariable analysis		Single variable analysis		Multivariable analysis		Single variable analysis		Multivariable analysis	
		OR (95% CI)	P*	aOR (95% CI)	*P*	OR (95% CI)	P*	aOR (95% CI)	*P*	OR (95% CI)	P*	aOR (95% CI)	*P*
Age group, years†	0–19	Ref.	**<0**·**001**	Ref.	—	Ref.	**<0·001**	Ref.	—	Ref.	**<0·001**	Ref.	—
	20–39	0·90 (0·34–2·39)	0·833	0·98 (0·36–2·67)	0·966	1·48 (0·62–3·52)	0·377	**1·77 (0·72–4·37)**	**0·215**	1·61 (0·58–4·44)	0·361	1·73 (0·59–5·07)	0·317
	40–59	**2·44 (1·21–4·93)**	**0·013**	2·04 (0·97–4·28)	0·060	**3·83 (1·94–7·60)**	**<0·001**	**3·09 (1·50–6·35)**	**0·002**	**5·44 (2·49–11·90)**	**<0·001**	**4·17 (1·81–9·62)**	**<0·001**
	60–79	**4·40 (2·30–8·44)**	**<0·001**	**2·96 (1·46–5·98)**	**0·003**	**8·89 (4·68–16·87)**	**<0·001**	**6·16 (3·11–12·20)**	**<0·001**	**16·76 (7·93–35·43)**	**<0·001**	**12·30 (5·55–27·26)**	**<0·001**
	⩾80	**9·77 (5·06–18·87)**	**<0·001**	**6·58 (3·20–13·50)**	**<0·001**	**20·77 (10·74–40·17)**	**<0·001**	**17·76 (8·71–36·19)**	**<0·001**	**60·14 (27·11–133·42)**	**<0·001**	**62·86 (26·54–148·90)**	**<0·001**
Sex†	Female	Ref.	—	Ref.	—	Ref.		Ref.	—	Ref.	—	Ref.	—
	Male	1·07 (0·81–1·41)	0·636	1·28 (0·94–1·75)	0·116	1·14 (0·89–1·45)	0·303	**1·64 (1·23–2·45)**	**0·001**	1·17 (0·89–1·53)	0·244	**1·96 (1·37–2·81)**	**<0·001**
Year of diagnosis	2006–2007	Ref.	0·542	—	—	Ref.	0·447	—	—	Ref.	0·460	—	—
	2007–2008	0·94 (0·59–1·23)	0·775	—	—	0·83 (0·55–1·23)	0·353	—	—	1·06 (0·73–1·56)	0·747	—	—
	2008–2009	0·64 (0·38–1·07)	0·090	—	—	0·72 (0·46–1·10)	0·131	—	—	0·75 (0·50–1·13)	0·169	—	—
	2009–2010	0·89 (0·56–1·44)	0·646	—	—	0·78 (0·52–1·19)	0·256	—	—	0·97 (0·65–1·43)	0·873	—	—
	2010–2011	0·81 (0·50–1·32)	0·326	—	—	0·74 (0·48–1·12)	0·156	—	—	0·81 (0·46–1·42)	0·458	—	—
	2011–2012	0·76 (0·45–1·31)	0·195	—	—	1·11 (0·71–1·72)	0·647	—	—	—	—	—	—
	2012–2013	0·62 (0·34–1·14)	0·124	—	—	0·98 (0·47–2·01)	0·949	—	—	—	—	—	—
Deprivation	Quintile 1	Ref	**0·050**	Ref.	—	Ref.	0·486	—	—	Ref.	0·065	Ref.	—
	Quintile 2	1·26 (0·74–2·15)	0·393	1·33 (0·75–2·37)	0·336	1·21 (0·78–1·89)	0·391	—	—	1·06 (0·64–1·75)	0·817	0·85 (0·44–1·63)	0·621
	Quintile 3	1·39 (0·82–2·33)	0·217	1·50 (0·85–2·64)	0·165	1·12 (0·72–1·73)	0·612	—	—	1·50 (0·93–2·43)	0·099	1·60 (0·85–3·00)	0·144
	Quintile 4	**1·97 (1·20–3·24)**	**0·007**	**2·30 (1·34–3·95)**	**0·003**	1·41 (0·92–2·15)	0·113	—	—	**1·75 (1·10–2·80)**	**0·019**	**2·18 (1·18–4·01)**	**0·012**
	Quintile 5	1·27 (0·77–2·10)	0·343	1·50 (0·87–2·60)	0·647	1·08 (0·71–1·63)	0·724	—	—	1·24 (0·78–1·97)	0·360	1·47 (0·80–2·68)	0·213
Immunization	Unvaccinated	Ref.	—	—	—	Ref.	—	—	—	Ref.	—	—	—
	Vaccinated	**1·85 (1·36–2·52)**	**<0·001**	—	—	**2·17 (1·69–2·79)**	**<0·001**	—	—	**2·77 (2·11–3·65)**	**<0·001**	—	—
Serotype	Other†	Ref.	**<0·001**	Ref.	—	Ref.	**<0·001**	—	—	Ref.	**<0·001**	Ref.	—
	1	**0·38 (0·16–0·90)**	**0·028**	0·81 (0·33–1·99)	0·643	**0·16 (0·08–0·34)**	**<0·001**	**0·30 (0·13–0·66)**	**0·003**	**0·17 (0·08–0·36)**	<0·001	**0·33 (0·13–0·80)**	**0·015**
	3	**2·24 (1·11–4·51)**	**0·024**	**2·20 (1·05–4·60)**	**0·036**	1·32 (0·73–2·38)	0·352	1·22 (0·64–2·34)	0·549	1·05 (0·51–2·16)	0·890	0·89 (0·37–2·18)	0·802
	4	1·00 (0·33–3·01)	1·000	1·22 (0·38–3·94)	0·742	0·41 (0·15–1·11)	0·079	0·43 (0·15–1·26)	0·124	**0·39 (0·15–1·04)**	0·060	0·31 (0·09–1·01)	0·051
	6A	**3·27 (1·33–8·07)**	**0·010**	**3·11 (1·19–8·17)**	**0·021**	**2·40 (1·06–5·43)**	**0·036**	2·10 (0·84–5·25)	0·113	1·70 (0·68–4·22)	0·253	1·42 (0·47–4·31)	0·533
	6B	2·21 (0·79–6·22)	0·133	2·12 (0·71–6·30)	0·177	2·00 (0·82–4·86)	0·126	1·63 (0·62–4·29)	0·327	1·56 (0·59–4·12)	0·367	1·16 (0·37–3·60)	0·796
	6C	**3·00 (1·20–7·52)**	**0·019**	2·33 (0·88–6·20)	0·089	**2·29 (1·09–5·99)**	**0·032**	1·70 (0·68–4·22)	0·249	1·22 (0·35–4·34)	0·751	0·89 (0·20–4·01)	0·884
	7F	0·77 (0·36–1·64)	0·493	1·12 (0·51–2·49)	0·776	**0·46 (0·24–0·86)**	**0·015**	0·62 (0·31–1·23)	0·168	0·50 (0·24–1·03)	0·060	0·66 (0·27–1·58)	0·347
	8	0·76 (0·33–1·77)	0·522	0·83 (0·35–2·00)	0·680	0·63 (0·32–1·16)	0·185	0·54 (0·26–1·12)	0·096	0·72 (0·34–1·55)	0·406	0·47 (0·19–1·17)	0·104
	9N	**4·15 (1·50**–**11·53)**	**0·006**	**4·48 (1·50–13·37)**	**0·007**	2·40 (0·93–6·21)	0·071	2·35 (0·82–6·70)	0·110	2·45 (0·79–7·60)	0·119	2·56 (0·65–10·20)	0·181
	9V	1·75 (0·63–4·83)	0·280	1·50 (0·52–4·35)	0·453	0·95 (0·40–2·28)	0·913	0·63 (0·24–1·64)	0·348	0·87 (0·34–2·19)	0·762	0·39 (0·13–1·16)	0·089
	10A	2·00 (0·56–7·09)	0·283	2·63 (0·66–10·42)	0·169	0·55 (0·14–2·11)	0·380	0·61 (0·13–2·81)	0·529	—	—	—	—
	11A	**3·43 (1·22–9·67)**	**0·020**	2·66 (0·90–7·91)	0·078	**2·89 (1·11–7·54)**	**0·030**	2·39 (0·84–6·80)	0·103	4·09 (1·00–16·72)	0·050	2·76 (0·55–13·89)	0·219
	12F	0·91 (0·30–2·72)	0·865	0·93 (0·30–2·91)	0·900	0·85 (0·37–1·95)	0·695	0·80 (0·32–1·98)	0·625	0·47 (0·17–1·26)	0·132	0·42 (0·13–1·32)	0·138
	14	1·24 (0·46–3·33)	0·676	1·12 (0·40–3·18)	0·825	1·20 (0·55–2·62)	0·647	0·67 (0·27–1·62)	0·370	1·23 (0·52–2·87)	0·636	0·64 (0·22–1·90)	0·427
	18C	0·86 (0·23–3·26)	0·821	0·79 (0·19–3·22)	0·738	0·67 (0·24–1·86)	0·439	0·61 (0·20–1·90)	0·399	0·70 (0·25–1·97)	0·502	0·68 (0·19–2·44)	0·555
	19A	1·63 (0·80–3·34)	0·182	1·55 (0·72–3·32)	0·259	0·67 (0·36–1·24)	0·198	0·52 (0·26–1·02)	0·058	0·74 (0·35–1·57)	0·428	0·48 (0·19–1·24)	0·129
	19F	**3·60 (1·32–9·80)**	**0·012**	**3·90 (1·32–11·49)**	**0·013**	**2·80 (1·11–7·07)**	**0·029**	**3·27 (1·13–9·43)**	**0·029**	1·84 (0·64–5·30)	0·258	2·27 (0·64–8·51)	0·225
	20	1·38 (0·35–5·49)	0·643	1·20 (0·28–5·07)	0·804	0·67 (0·20–2·25)	0·396	0·51 (0·14–1·84)	0·300	1·02 (0·27–3·80)	0·973	0·63 (0·14–2·80)	0·544
	22F	1·40 (0·35–5·46)	0·397	1·26 (0·56–2·83)	0·575	0·74 (0·39–1·43)	0·371	0·56 (0·27–1·16)	0·120	0·96 (0·44–2·07)	0·915	0·86 (0·34–2·19)	0·749
	23A	2·00 (0·63–6·38)	0·124	1·34 (0·40–4·55)	0·635	2·22 (0·81–6·07)	0·119	1·49 (0·49–4·52)	0·479	1·43 (0·42–4·88)	0·566	0·85 (0·19–3·78)	0·829
	23F	1·50 (0·55–4·09)	0·428	1·42 (0·49–4·08)	0·517	1·04 (0·46–2·39)	0·920	0·69 (0·28–1·72)	0·424	1·47 (0·61–3·57)	0·392	0·75 (0·25–2·19)	0·592
	31	2·00 (0·56–7·09)	0·283	1·59 (0·41–6·14)	0·499	0·73 (0·21–2·48)	0·611	0·40 (0·11–1·50)	0·176	1·64 (0·33–8·10	0·546	0·46 (0·08–2·67)	0·391
	33F	2·16 (0·84–5·58)	0·112	2·33 (0·84–6·49)	0·104	1·20 (0·52–2·79)	0·672	1·08 (0·42–2·79)	0·876	1·47 (0·54–4·05)	0·453	1·53 (0·43–5·45)	0·515
	35F	1·38 (0·35–5·49)	0·643	0·86 (0·20–3·64)	0·839	1·71 (0·53–5·57)	0·370	1·35 (0·36–5·04)	0·653	1·47 (0·40–5·48)	0·564	1·58 (0·30–8·34)	0·590
Clinical presentation	Other	Ref.	**0·005**	Ref.	—	Ref.	**0·008**	—	—	Ref.	0·007	—	—
	Meningitis	0·81 (0·44–1·51)	0·513	1·33 (0·67–2·67)	0·416	**0·44 (0·25–0·79)**	**0·006**	—	—	**0·41 (0·22–0·79)**	**0·007**	—	—
	Pneumonia	0·88 (0·57–1·35)	0·557	0·86 (0·53–1·40)	0·557	0·80 (0·55–1·15)	0·228	—	—	1·04 (0·69–1·55)	0·865	—	—
	Septicaemia	**1·86 (1·07–3·23)**	**0·027**	1·70 (0·92–3·14)	0·091	1·19 (0·72–1·98)	0·497	—	—	1·28 (0·69–2·37)	0·434	—	—
Alcohol misuse	No	Ref.	—	—	—	Ref.	—	—	—	Ref.	—	—	—
	Yes	1·01 (0·61–1·66)	0·973	—	—	0·76 (0·48–1·20)	0·240	—	—	0·91 (0·55–1·48)	0·692	—	—
Chronic heart disease	No	Ref.	—	—	—	Ref.	—	—	—	Ref.	—	Ref.	—
	Yes	**2·61 (1·93–3·53)**	**<0·001**	—	—	**3·23 (2·45–4·28)**	**<0·001**	—	—	**4·11 (2·95–5·73)**	**<0·001**	**1·69 (1·11–2·57)**	**0·014**
Chronic liver disease	No	Ref.	—	—	—	Ref.	—	—	—	Ref.	—	Ref.	—
	Yes	**1·89 (1·06–3·39)**	**0·032**	—	—	1·63 (0·94–2·84)	0·084	—	—	1·82 (0·93–3·55)	0·079	**3·34 (1·47–7·63)**	**0·004**
Chronic lung disease	No	Ref.	—	—	—	Ref.	—	—	—	Ref.	—	Ref.	—
	Yes	**1·72 (1·27–2·32)**	**<0·001**	—	—	**1·89 (1·44–2·47)**	**<0·001**	—	—	**2·79 (2·05–3·81)**	**<0·001**	**2·08 (1·39–3·12)**	**<0·001**
Chronic renal disease	No	Ref.	—	—	—	Ref.	—	Ref.	—	Ref.	—	Ref.	—
	Yes	**1·90 (1·28–2·82)**	**0·002**	—	—	**1·84 (1·27–2·68)**	**0·001**	**0·61 (0·38–0·99)**	**0·048**	**1·85 (1·20–2·84)**	**0·005**	**0·55 (0·31–0·96)**	**0·036**
Diabetes	No	Ref.	—	—	—	Ref.	—	—	—	Ref.	—	—	—
	Yes	1·45 (0·97–2·17)	0·072	—	—	**1·49 (1·04–2·15)**	**0·031**	—	—	1·53 (1·01–2·34)	0·046	—	—
Immuno- suppression	No	Ref.	—	—	—	Ref.	—	Ref.	—	Ref.	—	Ref.	—
	Yes	**1·67 (1·12–2·27)**	**0·012**	—	—	**2·94 (2·06–4·23)**	**<0·001**	**2·44 (1·57–3·81)**	**<0·001**	**2·77 (1·77–4·34)**	**<0·001**	**3·53 (2·01–6·19)**	**<0·001**
No. of risk factors	0	Ref.	**<0·001**	Ref.	—	Ref.	**<0·001**	Ref.	—	Ref.	**<0·001**	—	—
	1	**2·89 (2·03–4·12)**	**<0·001**	**2·11 (1·43–3·13)**	**<0·001**	**2·78 (2·05–3·80)**	**<0·001**	**1·64 (1·14–2·36)**	**0·008**	**3·41 (2·46–4·74)**	**<0·001**	—	—
	⩾2	**3·63 (2·53–5·23)**	**<0·001**	**2·17 (1·43–3·31)**	**0·019**	**4·73 (3·43–6·52)**	**<0·001**	**2·22 (1·43–3·43)**	**<0·001**	**5·82 (4·05–8·37)**	**<0·001**	—	—

aOR, Adjusted odds ratio; CI, confidence interval; Ref., reference group.

Values in bold indicate statistical significance (*P*<0·05).

* For reference groups the Wald test *P* value is reported.

† Included in the multivariable model irrespective of significance.

**Fig. 3.**
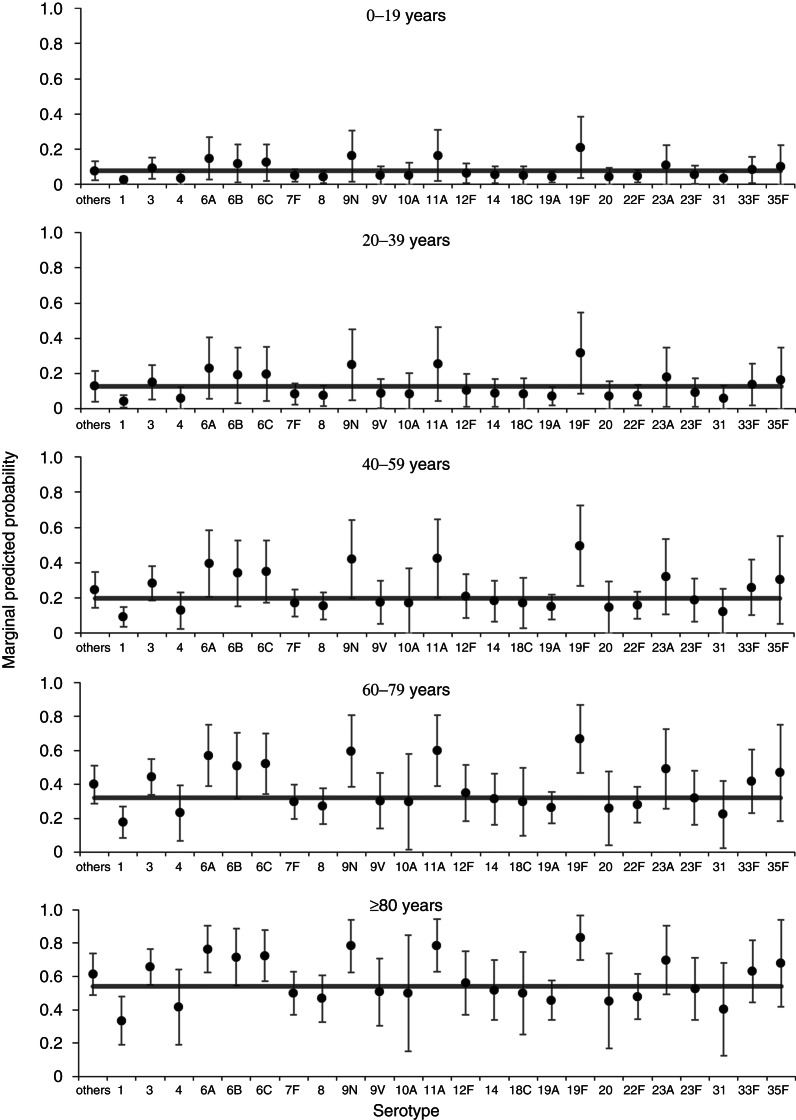
Predicted marginal probabilities of survival after 12 months following diagnosis with invasive pneumococcal disease by serotype. The grey line indicates the average for each age group. Probabilities are adjusted for sex, serotype and risk factors (number of, chronic renal disease, and immunosuppression).

After extending the all-cause mortality window to 12 months, one serotype remained associated with increased odds of mortality by this time point (19 F) and one serotype (1) was associated with reduced odds of mortality, both compared to the group of other serotypes (Table 3). Nonetheless, there remained substantial variation in predicted 12-month all-cause mortality rates with different serotypes for older age groups (Fig. 3). When comparing significant predictors to the 30-day all-cause mortality model, deprivation and clinical presentation were no longer significant predictors of death, while immunosuppression was now associated of increased odds of death and chronic renal disease was associated with reduced odds of death (Supplementary Table S2, Table 3). The 12-month mortality model also showed an acceptable goodness of fit (*χ*^2^ = 7·49, d.f. = 8, *P* = 0·485), no reduction in fit compared to the full starting model (*χ*^2^ = 5·17, d.f. = 7, *P* = 0·640), and a good classification rate for cases (77%).

**Fig. 4.**
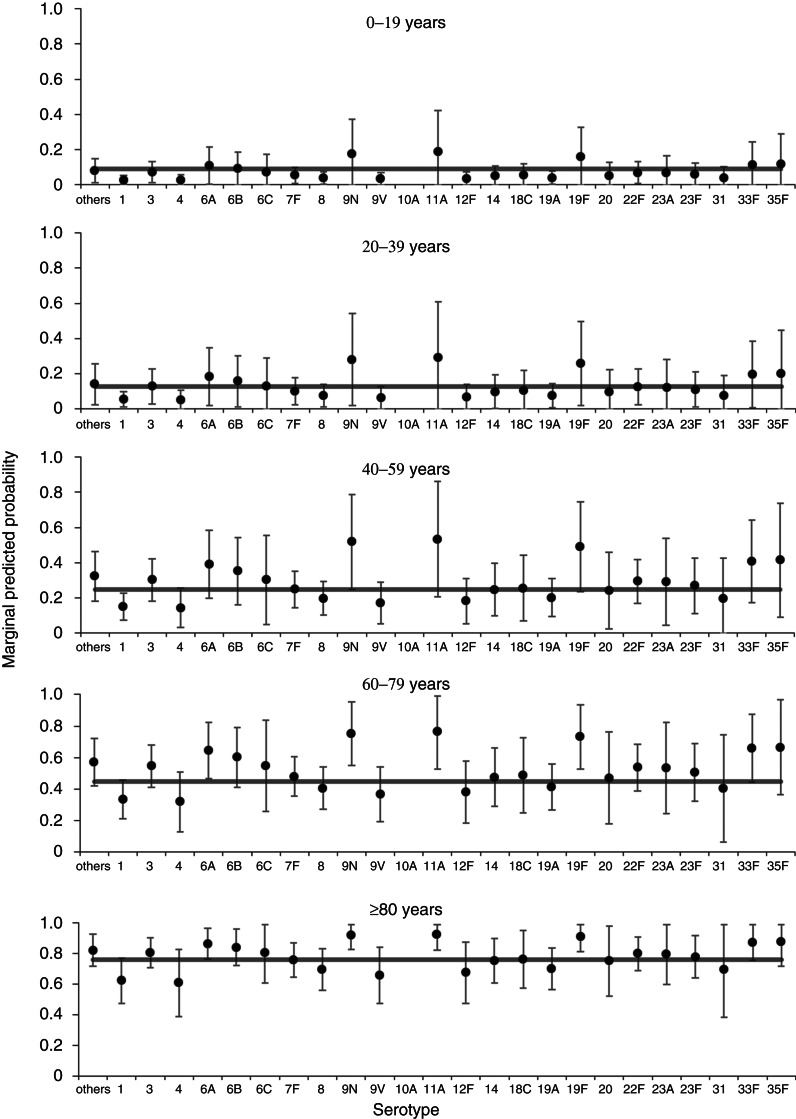
Predicted marginal probabilities of survival after 36 months following diagnosis with invasive pneumococcal disease by serotype. The grey line indicates the average for each age group. Probabilities are adjusted for sex, pneumococcal vaccination status, deprivation, serotype, and risk factors (chronic liver disease, chronic lung disease, and immunosuppression).

Extending the mortality window to 36 months further reduced the association of serotype with mortality (Table 3). Only one individual serotype (1) remained a significant predictor of (a reduced) all-cause mortality at 36 months compared to the group of other serotypes. For this model, the number of IPD risk factors was no longer a significant predictor, but three additional individual risk factors (chronic heart disease, chronic liver disease, chronic lung disease, plus immunosuppression also included in the 12-month model) were now associated with increased odds of death (Supplementary Table S3, Table 3). Similarly to the 30-day model (but not the 12-month model), deprivation was a significant predictor of all-cause mortality within 36 months. As with both other time-point models there was substantial variation in predicted all-cause mortality rates with different serotypes for older age groups (Fig. 4). The 36-month mortality model also showed an acceptable goodness of fit (*χ*^2^ = 5·20, d.f. = 8, *P* = 0·736), no reduction in fit compared to the full starting model (*χ*^2^ = 5·07, d.f. = 7, *P* = 0·651), and a good classification rate for cases (78%).

Sensitivity analysis of all-cause mortality by time points showed no pattern or substantial change in crude odds ratios when using all available cases and not just cases without missing data: 30-day (median +18% change, range −119% to +26%); 12 months (median −8% change, range −80% to +99%); 36 months (median −10% change, range −62% to +9%). Given the small numbers of cases for certain serotypes, changes in the significance of associations were found, although there was no trend associated with the sensitivity analysis (Supplementary Table S4). Using all available data for final multivariable models did not lead to the loss of any significant associations for individual serotypes (Supplementary Table S5).

### Analysis of longer-term survival

**Fig. 5.**
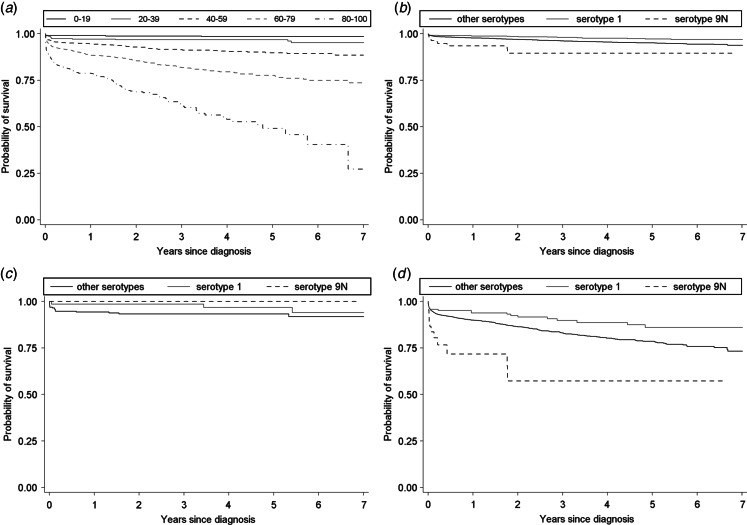
Adjusted Kaplan–Meier survival curves for time since diagnosis of all cases of invasive pneumococcal disease. (*a*) All serotypes by age group, (*b*) significantly associated serotypes for all ages, (*c*) significantly associated serotypes for ages 0–39 years, (*d*) significantly associated serotypes for ages ⩾40 years. Survival function is adjusted within each age group for sex, deprivation quintile, clinical presentation, chronic heart disease, chronic liver disease, chronic lung disease, and immunosuppression. Only the two serotypes significantly associated with survival from the multivariable model are shown individually.

Of the 1316 cases included in the analysis, 542 (41·2%, 95% CI 38·5–43·9) died within the available follow-up period (from date of diagnosis to 30 September 2013; median 889 days, range 1–2557). For the all cases model, the main-effects Cox PH model included age group, sex, deprivation, serotype, clinical presentation, chronic heart disease, chronic liver disease, chronic lung disease, and immunosuppression (Supplementary Table S6, Table 4). Sensitivity analysis using all available cases rather than just cases without any missing data suggested no substantial change in crude associations if all available data were included (Supplementary Table S4). The assumption of PH was not met for three age groups (40–59, 60–79, ⩾80 years), sex, one serotype (3), one clinical presentation (meningitis) and immunosuppression. Subsequent time-dependent effects were significant (*P* < 0·05) for all three age groups, sex, clinical presentation (meningitis) and immunosuppression (Supplementary Table S6). The extended Cox PH model represented an overall significantly improved fit compared to the main-effects model (*χ*^2^ = 55·99, d.f. = 6, *P* < 0·001) and the results of this model were used for assessment of the hazard ratio (HR) (Table 4). Two serotypes (1, reduced mortality; 9N, increased mortality) had statistically significant HRs, demonstrating serotype-specific variation in survival following infection, after adjustment for other significant predictors. Those average serotype-specific effects are restricted to older adults (Fig. 5). There was no change in the significance of main-effects predictors for serotypes using all available data for the final multivariable model (Supplementary Table S5).

**Table 4.** Crude and adjusted associations with longer term survival following diagnosis with invasive pneumococcal disease

		All cases						Cases surviving >30 days post-diagnosis			
Variable	Category	Log rank test		Cox proportional hazards model				Cox proportional hazards model			
				Main effect		Time-dependent effect		Main effect		Time-dependent effect	
		*χ*^2^ (d.f.)	*P*	HR (95% CI)	P*	HR (95% CI)	*P*	HR (95% CI)	P*	HR (95% CI)	*P*
Age group, years†	0–19	**396·55** (**4)**	**<0**·**001**	Ref.	**<0·001**	—	—	Ref.	**<0·001**	—	—
	20–39	—	—	1·83 (0·87–3·88)	0·114	—	—	**6·96 (1·46–33·12)**	**0·015**	—	—
	40–59	—	—	1·93 (0·90–4·13)	0·091	**1·26 (1·06–1·49)**	**0·008**	**15·06 (3·61–62·83)**	**<0·001**	—	—
	60–79	—	—	**2·49 (1·23–5·06)**	**0·011**	**1·41 (1·20–1·65)**	**<0·001**	**38·00 (9·28–155·66)**	**<0·001**	—	—
	⩾80	—	—	**4·60 (2·25–9·39)**	**<0·001**	**1·48 (1·26–1·74)**	**<0·001**	**96·24 (23·27–397·94)**	**<0·001**	—	—
Sex†	Female	1·69 (1)	0·193	Ref.	—	—	—	Ref.	—	—	—
	Male	—	—	1·18 (0·88–1·60)	0·268	—	—	**1·82 (1·42–2·33)**	**<0·001**	—	—
Year of diagnosis	2006–2007	4·49 (6)	0·611	—	—	—	—	—	—	—	—
	2007–2008	—	—	—	—	—	—	—	—	—	—
	2008–2009	—	—	—	—	—	—	—	—	—	—
	2009–2010	—	—	—	—	—	—	—	—	—	—
	2010–2011	—	—	—	—	—	—	—	—	—	—
	2011–2012	—	—	—	—	—	—	—	—	—	—
	2012–2013	—	—	—	—	—	—	—	—	—	—
Deprivation	Quintile 1	**12·11 (4)**	**0·017**	Ref.	**0·006**	—	—	—	—	—	—
	Quintile 2	—	—	1·01 (0·73–1·40)	0·956	—	—	—	—	—	—
	Quintile 3	—	—	1·27 (0·93–1·75)	0·138	—	—	—	—	—	—
	Quintile 4	—	—	**1·60 (1·18–2·17)**	**0·003**	—	—	—	—	—	—
	Quintile 5	—	—	1·27 (0·93–1·72)	0·135	—	—	—	—	—	—
Immunization	Vaccinated	**65·17 (1)**	**<0·001**	—	—	—	—	—	—	—	—
	Unvaccinated	—	—	—	—	—	—	—	—	—	—
Serotype	Other†	**157·50 (24)**	**<0·001**	Ref.	**0·002**	—	—	Ref.	**<0·001**	—	—
	1	—	—	**0·51 (0·30–0·86)**	**0·011**	—	—	**0·32 (0·16–0·63)**	**0·001**	—	—
	3	—	—	1·08 (0·73–1·61)	0·700	—	—	0·65 (0·38–1·11)	0·117	—	—
	4	—	—	0·77 (0·40–1·48)	0·432	—	—	0·50 (0·22–1·17)	0·109	—	—
	6A	—	—	1·34 (0·77–2·30)	0·297	—	—	0·76 (0·34–1·70)	0·505	—	—
	6B	—	—	1·33 (0·74–2·38)	0·346	—	—	0·82 (0·38–1·76)	0·610	—	—
	6C	—	—	1·21 (0·70–2·09)	0·487	—	—	0·80 (0·36–1·75)	0·577	—	—
	7F	—	—	0·68 (0·43–1·09)	0·107	—	—	**0·43 (0·23–0·80)**	**0·008**	—	—
	8	—	—	0·81 (0·52–1·27)	0·357	—	—	0·67 (0·39–1·15)	0·150	—	—
	9N	—	—	**2·37 (1·29–4·37)**	**0·007**	—	—	1·74 (0·67–4·56)	0·257	—	—
	9V	—	—	0·95 (0·55–1·65)	0·864	—	—	0·60 (0·30–1·19)	0·147	—	—
	10A	—	—	0·96 (0·34–2·71)	0·942	—	—	<0·1 (0·00–.)	1·000	—	—
	11A	—	—	1·68 (0·96–2·93)	0·067	—	—	1·29 (0·63–2·68)	0·487	—	—
	12F	—	—	0·73 (0·39–1·35)	0·311	—	—	0·51 (0·23–1·12)	0·093	—	—
	14	—	—	0·92 (0·55–1·55)	0·765	—	—	0·69 (0·37–1·29)	0·244	—	—
	18C	—	—	1·25 (0·62–2·50)	0·530	—	—	1·53 (0·66–3·54)	0·324	—	—
	19A	—	—	0·80 (0·53–1·22)	0·305	—	—	**0·45 (0·26–0·80)**	**0·007**	—	—
	19F	—	—	1·64 (0·91–2·97)	0·100	—	—	0·98 (0·42–2·31)	0·971	—	—
	20	—	—	0·70 (0·31–1·57)	0·390	—	—	0·42 (0·15–1·22)	0·112	—	—
	22F	—	—	0·83 (0·54–1·27)	0·387	—	—	**0·57 (0·33–0·98)**	**0·044**	—	—
	23A	—	—	1·33 (0·72–2·44)	0·358	—	—	1·17 (0·55–2·49)	0·679	—	—
	23F	—	—	0·70 (0·41–1·21)	0·202	—	—	**0·47 (0·24–0·91)**	**0·024**	—	—
	31	—	—	0·96 (0·48–1·93)	0·908	—	—	0·62 (0·25–1·52)	0·294	—	—
	33F	—	—	1·31 (0·76–2·26)	0·328	—	—	0·86 (0·43–1·74)	0·675	—	—
	35F	—	—	0·89 (0·42–1·90)	0·763	—	—	**26·76 (4·77–149·58)**	**<0·001**	—	—
Clinical presentation	Other	**27·32 (3)**	**<0·001**	Ref.	**0·039**	—	—	Ref.	—	—	—
	Meningitis	—	—	1·25 (0·69–2·26)	0·469	**0·78 (0·67–0·91)**	**0·002**	**0·20 (0·09–0·46)**	**<0·001**	—	—
	Pneumonia	—	—	0·77 (0·59–1·00)	0·052	—	—	0·73 (0·51–1·03)	0·072	—	—
	Septicaemia	—	—	1·02 (0·72–1·45)	0·925	—	—	0·66 (0·40–1·11)	0·121	—	—
Alcohol misuse	No	0·01 (1)	0·916	—	—	—	—	—		—	—
	Yes	—	—	—	—	—	—	—		—	—
Chronic heart disease	No	**132·46 (1)**	**<0·001**	Ref.	—	—	—	Ref.	—	—	—
	Yes	—	—	**1·33 (1·10–1·61)**	**0·004**	—	—	**1·36 (1·05–1·77)**	**0·019**	—	—
Chronic liver disease	No	**16·59 (1)**	**<0·001**	Ref.	—	—	—	Ref.	—	—	—
	Yes	—	—	**2·30 (1·62–3·27)**	**<0·001**	—	—	**2·88 (1·78–4·65)**	**<0·001**	—	—
Chronic lung disease	No	**55·39 (1)**	**<0·001**	Ref.	—	—	—	Ref.	—	—	—
	Yes	—	—	**1·29 (0·85–1·96)**	**0·008**	—	—	1·37 (1·06–1·77)	0·015	—	—
Chronic renal disease	No	**23·77 (1)**	**<0·001**	—	—	—	—	—	—	—	—
	Yes	—	—	—	—	—	—	—	—	—	—
Diabetes	No	**4·52 (1)**	**0·033**	—	—	—	—	—	—	—	—
	Yes	—	—	—	—	—	—	—	—	—	—
Immunosuppression	No	**63·00 (1)**	**<0·001**	Ref.	—	—	—	Ref.	—	—	—
	Yes	—	—	1·29 (0·85–1·96)	0·229	**1·11 (1·02–1·22)**	**0·020**	**2·53 (1·87–3·42)**	**<0·001**	—	—
Number of risk factors	0	**193·09 (2)**	**<0·001**	—	—	—	—	—	—	—	—
	1	—	—	—	—	—	—	—	—	—	—
	⩾2	—	—	—	—	—	—	—	—	—	—

CI, confidence interval; d.f., degrees of freedom; HR, hazard ratio; Ref., reference group.

Values in bold indicate statistical significance (*P*<0·05).

* For reference groups the Wald test *P* value is reported.

† Included in the multivariable model irrespective of significance.

The analysis using an observation period starting 30 days after diagnosis included 1073 cases, of which 299 (27·9%, 95% CI 25·2–30·7) died within the available follow-up period (median 1140 days, range 1–2527). The Cox PH model included main effects for age group, sex, serotype, clinical presentation, chronic heart disease, chronic liver disease, chronic lung disease and immunosuppression (Supplementary Table S5, Table 4). The assumption of PH was not met for two serotypes (1, 35 F) and chronic liver disease. Subsequent time-dependent effects were significant (*P* < 0·05) only for serotype 35 F (Supplementary Table S5). The extended Cox PH model represented an overall significantly improved fit compared to the main-effects model (*χ*^2^ = 4·81, d.f. = 1, *P* = 0·028) and the results of this model were used for assessment of the HR (Table 4). Six serotypes (1, 7 F, 19A, 22 F, 23 F, 35 F) had statistically significant HRs, although only one of these (35 F) was for an increased risk of all-cause mortality. The association between serotype 9N and increased all-cause mortality found in the model containing the whole observation period (Table 3) was not found when excluding the first 30 days following diagnosis from the observation period (and thus removing from analysis the 243 cases that died within that time period).

## DISCUSSION

Given the requirement to demonstrate the cost-effectiveness of vaccination programmes, together with the need to select only certain serotypes for inclusion in vaccine formulations, the existence of serotype-specific mortality has implications for determining the societal benefits of pneumococcal vaccines. It is well-established that mortality rates immediately following IPD are associated with the serotype of *S. pneumoniae* causing the infection [8]. This is, to our knowledge, the first study to consider the potential association between serotype and longer-term mortality. Using a cohort of IPD cases from a region of England with a population of ~2·5 million persons, we have estimated the relative associations between short-term (30-day) and long-term (⩽7 years) all-cause mortality following IPD and 24 different serotypes of *S. pneumoniae.*

Our findings support those of previous studies that pneumococcal serotype is independently associated with differential 30-day mortality rates following IPD. After adjusting for confounding by the effects of the living environment (deprivation), comorbidities, and clinical presentation, four serotypes were associated with increased mortality rates at 30 days (3, 6A, 9N, 19 F). These associations are supported by a meta-analysis and systematic review of other studies, including assessment of individual studies with a variety of reference groups, effect sizes and strengths of association [7, 8]. Due to the reduced statistical power for children, the statistical significance of associations between serotype and 30-day mortality is restricted to older adults. When the window following infection was widened to 12 months, only one serotype (19 F) remained associated with increased mortality. At 36 months, no associations between individual serotypes and increased mortality were found. Statistically significant associations were found for reduced mortality rates associated with serotype 1 (12 and 36 months). This finding is supported by considerable existing evidence for reduced virulence and relative mortality for serotype 1, although the mechanism of this reduced severity has yet to be established [3, 7, 8].

The lack of association between three serotypes (3, 6A, 9N) and increased mortality rates using time-frames beyond 30 days suggests that the clinical effects of differential virulence due to these serotypes are restricted to pathogenesis (and/or response to treatment) during the acute stage of the infection. Serotype 19 F was found to be associated with increased mortality at 12 months as well as 30 days, but not when the window was expanded to 36 months. Furthermore, mortality during the 30 days following infection was significantly associated with the cumulative number of IPD risk factors, rather than individual risk factors being themselves associated with increased mortality, as is seen with the 12 months (immunosuppression) and 36 months (chronic heart disease, chronic liver disease, chronic lung disease, immunosuppression) analysis. It would appear that there is a switch from the combined effect of multiple comorbidities (within 30 days and 12 months of infection) to an effect of specific comorbidities as the time window since infection is expanded, such that within the 36-month time period the number of risk factors is no longer itself significantly associated with mortality. The association between reduced mortality at 12 and 36 months and chronic renal disease may reflect increased contact rates with health services that minimizes the probability of late diagnosis associated with poorer outcomes.

Through the use of a multivariable Cox PH model we have produced fully adjusted, independent associations between serotype and longer-term mortality following IPD. As was seen with the longer window time-point analyses, specific risk factors are predictive of increased mortality rates, rather than the cumulative number. Over a time period of ⩽7 years, we have shown that serotype is associated with significant variation in mortality with one serotype associated with significantly reduced mortality (1) and one serotype associated with significantly increased mortality (9N). Due to a limited statistical power, these effects are only observable in older adults and higher-powered further studies are required to investigate these associations for children and younger adults. Both serotypes 1 and 9N have been previously shown to be associated with decreased and increased mortality, respectively [7, 8].

When survival analysis was limited to an observation period that excluded the first 30 days following diagnosis, an altered pattern of serotype-specific mortality rates was observed, with a greater number of serotypes associated with significantly lower mortality rates (serotypes 1, 7 F, 19A, 22 F, 23 F) and a different serotype associated with significantly higher mortality rates (serotype 35 F). However, the estimate for serotype 35 F has quite low precision and this statistical uncertainty requires further investigation with a larger dataset. Although the association between serotype 9N and increased mortality is no longer statistically significant, the point estimate for the HR remains >1 and the lack of significance may be due to the reduced power of the analysis compared to the full survival analysis.

We have not been able to consider the influence of events which have occurred post-diagnosis with IPD on the mortality of cases. Of course, it is expected that factors which might contribute towards an increased risk of mortality will have occurred in the follow-up period. However, for these factors to confound relative associations between mortality and individual serotypes they would need to be both causes of increased mortality and be associated with individual serotypes, and it is difficult to foresee situations when this would occur. Furthermore, it should be considered that this study has only assessed relative associations between serotypes and mortality following IPD; no inferences can be made regarding mortality following infection with individual serotypes compared to mortality for similar individuals who have not had IPD.

This study provides the first evidence that the pneumococcal serotype causing IPD is associated with differential longer-term mortality. Due to limited statistical power for children and younger adults it is currently not clear whether these associations apply to these age groups as well as to older adults. Further studies are required to determine the influence of serotype-specific longer-term mortality on the burden of IPD and the consequent implications for the relative benefit of different vaccine compositions.

## Supplementary material

For supplementary material accompanying this paper visit https://doi.org/10.1017/S0950268816000856. click here to view supplementary material
