# Supplementary material for: Serotype-specific differences in short- and longer-term mortality following invasive pneumococcal disease
Source: Epidemiol Infect. 2016 May 19;144(12):2654–69. doi: 10.1017/S0950268816000856 (PMC4988273; doi:10.1017/S0950268816000856)
Supplement: Supplementary file 1 [file S0950268816000856sup001.docx]

## Supplementary Table S1. Model building for the multivariable logistic regression model for 30-day survival.

| Model | Vs. | lnL | lnL statistic (P) | Variables (P) | Removed |
| --- | --- | --- | --- | --- | --- |
| M1 | - | -534.215 | - | Age group (<0.001), sex (0.138), deprivation (0.031), vaccination (0.367), serotype (0.073), clinical presentation (0.033), chronic heart disease (0.471), chronic liver disease (0.088), chronic lung disease (0.676), chronic renal disease (0.585), **diabetes (0.873)**, immunosuppression (0.259), number of risk factors (0.073) | - |
| M2 | M1 | -534.227 | 0.03 (0.8734) | Age group (<0.001), sex (0.136), deprivation (0.031), vaccination (0.371), serotype (0.073), clinical presentation (0.034), chronic heart disease (0.388), chronic liver disease (0.066), chronic lung disease (0.575), **chronic renal disease (0.600)**, immunosuppression (0.173), number of risk factors (0.074) | Diabetes |
| M3 | M2 | -534.365 | 0.28 (0.5997) | Age group (<0.001), sex (0.140), deprivation (0.032), vaccination (0.379), serotype (0.077), clinical presentation (0.033), chronic heart disease (0.264), chronic liver disease (0.051), **chronic lung disease (0.428)**, immunosuppression (0.103), number of risk factors (0.076) | Chronic renal disease |
| M4 | M3 | -534.681 | 0.63 (0.4267) | Age group (<0.001), sex (0.145), deprivation (0.026), vaccination (0.353), serotype (0.084), clinical presentation (0.041), **chronic heart disease (0.380),** chronic liver disease (0.070), chronic renal disease (0.617), immunosuppression (0.143), number of risk factors (0.038) | Chronic lung disease |
| M5 | M4 | -535.066 | 0.77 (0.3799) | Age group (<0.001), sex (0.128), deprivation (0.024), **vaccination (0.334)**, serotype (0.080), clinical presentation (0.037), chronic liver disease (0.088), number of risk factors (0.009) | Chronic heart disease |
| M6 | M5 | -535.534 | 0.93 (0.3336) | Age group (<0.001), sex (0.129), deprivation (0.027), serotype (0.080), clinical presentation (0.037), chronic liver disease (0.094), **immunosuppression (0.226)**, number of risk factors (0.003) | Vaccination |
| M7 | M6 | -536.255 | 1.44 (0.2298) | Age group (<0.001), sex (0.106), deprivation (0.030), serotype (0.080), clinical presentation (0.031), **chronic liver disease (0.129)**, number of risk factors (0.007) | Immunosuppression |
| M8 | M7 | -537.365 | 2.22 (0.1362) | Age group (<0.001), sex (0.116), deprivation (0.027), serotype (0.083), clinical presentation (0.028), number of risk factors (0.001) | Chronic liver disease |

lnL: log likelihood.

## Supplementary Table S2. Model building for the multivariable logistic regression model for 12-month survival.

| Model | Vs. | lnL | lnL statistic (P) | Variables (P) | Removed |
| --- | --- | --- | --- | --- | --- |
| M1 | - | -584.245 | - | Age group (<0.001), sex (0.001), vaccination (0.284), serotype (0.002), clinical presentation (0.676), chronic heart disease (0.324), **chronic lung disease (0.978)**, chronic renal disease (0.093), diabetes (0.519), immunosuppression (0.001), number of risk factors (0.307) | - |
| M2 | M1 | -584.246 | 0.00 (0.978) | Age group (<0.001), sex (0.001), vaccination (0.281), serotype (0.002), **clinical presentation (0.675)**, chronic heart disease (0.255), chronic renal disease (0.065), diabetes (0.466), immunosuppression (<0.001), number of risk factors (0.114) | Chronic lung disease |
| M3 | M2 | -585.004 | 1.52 (0.678) | Age group (<0.001), sex (0.001), vaccination (0.299), serotype (<0.001), chronic heart disease (0.250), chronic renal disease (0.075), diabetes (0.464), immunosuppression (<0.001), number of risk factors (0.106) | Clinical presentation |
| M4 | M3 | -585.274 | 0.54 (0.463) | Age group (<0.001), sex (0.001), **vaccination (0.301)**, serotype (<0.001), chronic heart disease (0.197), chronic renal disease (0.092), immunosuppression (<0.001), number of risk factors (0.139) | Diabetes |
| M5 | M4 | -585.807 | 1.07 (0.302) | Age group (<0.001), sex (0.001), serotype (<0.001), **chronic heart disease (0.187)**, chronic renal disease (0.093), immunosuppression (<0.001), number of risk factors (0.083) | Vaccination |
| M6 | M5 | -586.028 | 1.71 (0.190) | Age group (<0.001), sex (0.001), serotype (<0.001), chronic renal disease (0.048), immunosuppression (<0.001), number of risk factors (0.001) | Chronic heart disease |

lnL: log likelihood.

## Supplementary Table S3. Model building for the multivariable logistic regression model for 36-month survival.

| Model | Vs. | lnL | lnL statistic (P) | Variables (P) | Removed |
| --- | --- | --- | --- | --- | --- |
| M1 | - | -406.675 | - | Age group (<0.001), sex (<0.001), deprivation (0.008), vaccination (0.093), serotype (0.035), **clinical presentation (0.847)**, chronic heart disease (0.216), chronic liver disease (0.028), chronic lung disease (0.094), chronic renal disease (0.082), diabetes (0.599), immunosuppression (0.005), number of risk factors (0.659) | - |
| M2 | M1 | -407.088 | 0.83 (0.843) | Age group (<0.001), sex (<0.001), deprivation (0.007), vaccination (0.066), serotype (0.034), chronic heart disease (0.214), chronic liver disease (0.024), chronic lung disease (0.081), chronic renal disease (0.084), diabetes (0.628), immunosuppression (0.003), **number of risk factors (0.633)** | Clinical presentation |
| M3 | M2 | -407.544 | 0.91 (0.634) | Age group (<0.001), sex (<0.001), deprivation (0.007), vaccination (0.074), serotype (0.035), chronic heart disease (0.028), chronic liver disease (0.005), chronic lung disease (0.002), chronic renal disease (0.027), **diabetes (0.578)**, immunosuppression (<0.001) | Number of risk factors |
| M4 | M3 | -407.699 | 0.31 (0.578) | Age group (<0.001), sex (<0.001), deprivation (0.007), **vaccination (0.082)**, serotype (0.037), chronic heart disease (0.033), chronic liver disease (0.005), chronic lung disease (0.002), chronic renal disease (0.024), immunosuppression (<0.001) | Diabetes |
| M5 | M4 | -409.210 | 3.02 (0.082) | Age group (<0.001), sex (<0.001), deprivation (0.010), serotype (0.043), chronic heart disease (0.014), chronic liver disease (0.004), chronic lung disease (<0.001), chronic renal disease (0.036), immunosuppression (<0.001) | Vaccination |

lnL: log likelihood.

## Supplementary Table S4. Sensitivity analysis for single variable associations with mortality post-diagnosis with IPD.

| **Variable** | **Category** | **30-day survival** | | | | | **12-month survival** | | | | | **36-month survival** | | | | | **Longer-term survival (<7 years)** | | | |
| --- | --- | --- | --- | --- | --- | --- | --- | --- | --- | --- | --- | --- | --- | --- | --- | --- | --- | --- | --- | --- |
|  |  | **Complete cases only** | | **Full dataset** | | **% Δ OR** | **Complete cases only** | | **Full dataset** | | **% Δ OR** | **Complete cases only** | | **Full dataset** | | **% Δ OR** | **Complete cases only** | | **Full dataset** | |
|  |  | **OR_UN_ (95% CI)** | **P** | **OR_UN_ (95% CI)** | **P** |  | **OR_UN_ (95% CI)** | **P** | **OR_UN_ (95% CI)** | **P** |  | **OR_UN_ (95% CI)** | **P** | **OR_UN_ (95% CI)** | **P** |  | **Chi^2^ (DF)** | **P** | **Chi^2^ (DF)** | **P** |
| Age group† | 0-19 | Ref. | - | Ref. | - | - | Ref. | - | Ref. | - | - | Ref. | **<0.001** | Ref. | - | - | **396.55 (4)** | **<0.001** | **469.45 (4)** | **<0.001** |
|  | 20–39 | 0.90 (0.34–2.39) | 0.833 | 0.82 (0.31–2.12) | 0.676 | -10 | 1.48 (0.62–3.52) | 0.377 | **1.33 (0.57–3.12)** | **0.502** | -11 | 1.61 (0.58–4.44) | 0.361 | 1.29 (0.49–3.78) | 0.609 | -25 | - | - | - | - |
|  | 40–59 | **2.44 (1.21­–4.93)** | **0.013** | **2.51 (1.30–4.86)** | **0.006** | +3 | **3.83 (1.94–7.60)** | **<0.001** | **3.77 (1.98–7.18)** | **<0.001** | -2 | **5.44 (2.49–11.90)** | **<0.001** | **4.82 (2.38–9.77)** | **<0.001** | -13 | - | - | **-** | **-** |
|  | 60–79 | **4.40 (2.30–8.44)** | **<0.001** | **5.22 (2.82–9.67)** | **<0.001** | +16 | **8.89 (4.68–16.87)** | **<0.001** | **10.27 (5.57–18.92)** | **<0.001** | +13 | **16.76 (7.93–35.43)** | **<0.001** | **15.91 (8.10–31.27)** | **<0.001** | -5 | - | - | **-** | **-** |
|  | ≥80 | **9.77 (5.06–18.87)** | **<0.001** | **10.77 (5.77–20.10)** | **<0.001** | +9 | **20.77 (10.74–40.17)** | **<0.001** | **22.34 (11.93–41.85)** | **<0.001** | +7 | **60.14 (27.11–133.42)** | **<0.001** | **55.32 (26.79–114.26)** | **<0.001** | -9 | - | - | **-** | **-** |
| Sex† | Female | Ref. | - | Ref. | - | - | Ref. | - | Ref. | - | - | Ref. | - | Ref. | - |  | 1.69 (1) | 0.193 | **5.24 (1)** | **0.022** |
|  | Male | 1.07 (0.81–1.41) | 0.636 | 1.17 (0.91–1.50) | 0.231 | +9 | 1.14 (0.89–1.45) | 0.303 | 1.24 (0.99–1.56) | 0.057 | +8 | 1.17 (0.89–1.53) | 0.244 | 1.17 (0.91–1.51) | 0.211 | +0 | - | - | - | - |
| Year of diagnosis | 2006–07 | Ref. | - | Ref. | - | - | Ref. | 0.447 | Ref. | - | - | Ref. | 0.460 | Ref. | - |  | 4.49 (6) | 0.611 | 3.83 (6) | 0.700 |
|  | 2007­–08 | 0.94 (0.59–1.23) | 0.775 | 0.80 (0.52–1.22) | 0.295 | -18 | 0.83 (0.55–1.23) | 0.353 | 0.72 (0.50–1.05) | 0.091 | -15 | 1.06 (0.73–1.56) | 0.747 | 0.88 (0.62–1.26) | 0.493 | -20 | - | - | - | - |
|  | 2008–09 | 0.64 (0.38–1.07) | 0.090 | **0.58 (0.37–0.92)** | **0.021** | -10 | 0.72 (0.46–1.10) | 0.131 | **0.65 (0.44–0.97)** | **0.035** | -11 | 0.75 (0.50-1.13) | 0.169 | **0.66 (0.45–0.96)** | **0.029** | -14 | - | - | - | - |
|  | 2009–10 | 0.89 (0.56–1.44) | 0.646 | 0.85 (0.55–1.31) | 0.457 | -5 | 0.78 (0.52–1.19) | 0.256 | 0.78 (0.53–1.14) | 0.199 | +0 | 0.97 (0.65–1.43) | 0.873 | 0.89 (0.62–1.28) | 0.537 | -9 | - | - | - | - |
|  | 2010­–11 | 0.81 (0.50–1.32) | 0.326 | 0.69 (0.45–1.07) | 0.098 | -17 | 0.74 (0.48–1.12) | 0.156 | **0.65 (0.45–0.96)** | **0.031** | -14 | 0.81 (0.46–1.42) | 0.458 | 0.79 (0.47–1.33) | 0.371 | -3 | - | - | - | - |
|  | 2011–12 | 0.76 (0.45–1.31) | 0.195 | 0.67 (0.42–1.07) | 0.095 | -13 | 1.11 (0.71–1.72) | 0.647 | 0.94 (0.63–1.39) | 0.749 | -18 | - | - | - | - | - | - | - | - | - |
|  | 2012–13 | 0.62 (0.34–1.14) | 0.124 | 0.72 (0.45–1.15) | 0.164 | +14 | 0.98 (0.47–2.01) | 0.949 | 1.13 (0.65–1.96) | 0.672 | +13 | - | - | - | - | - | - | - | - | - |
| Deprivation | Quintile 1 | Ref | - | Ref. | - | - | Ref. | 0.486 | Ref. | - | - | Ref. | 0.065 | Ref. | - | - | **12.11 (4)** | **0.017** | **8.05 (4)** | **0.090** |
|  | Quintile 2 | 1.26 (0.74–2.15) | 0.393 | 1.07 (0.67–1.72) | 0.772 | -18 | 1.21 (0.78–1.89) | 0.391 | 1.11 (0.74–1.68) | 0.590 | -9 | 1.06 (0.64–1.75) | 0.817 | 0.96 (0.60–1.54) | 0.880 | -10 | - | - | - | - |
|  | Quintile 3 | 1.39 (0.82–2.33) | 0.217 | 1.18 (0.74–1.86) | 0.488 | -18 | 1.12 (0.72–1.73) | 0.612 | 1.09 (0.74–1.63) | 0.658 | -3 | 1.50 (0.93–2.43) | 0.099 | 1.22 (0.78–1.92) | 0.390 | -23 | - | - | - | - |
|  | Quintile 4 | **1.97 (1.20–3.24)** | **0.007** | **1.67 (1.08–2.58)** | **0.021** | -18 | 1.41 (0.92–2.15) | 0.113 | 1.32 (0.90–1.94) | 0.160 | -7 | **1.75 (1.10–2.80)** | **0.019** | **1.51 (0.97–2.33)** | **0.066** | -16 | - | - | **-** | **-** |
|  | Quintile 5 | 1.27 (0.77–2.10) | 0.343 | 1.14 (0.74–1.76) | 0.559 | -11 | 1.08 (0.71–1.63) | 0.724 | 1.05 (0.72–1.53) | 0.795 | -3 | 1.24 (0.78–1.97) | 0.360 | 1.10 (0.71–1.70) | 0.661 | -13 | - | - | - | - |
| Immunisation | Vaccinated | Ref. | - | Ref. | - | - | Ref. | - | Ref. | - | - | Ref. | - | Ref. | - | - | **65.17 (1)** | **<0.001** | **83.65 (1)** | **<0.001** |
|  | Unvaccinated | **1.85 (1.36–2.52)** | **<0.001** | **2.07 (1.59–2.69)** | **<0.001** | +11 | **2.17 (1.69–2.79)** | **<0.001** | **2.31 (1.82–2.92)** | **<0.001** | +6 | **2.77 (2.11–3.65)** | **<0.001** | **2.77 (2.12–3.62)** | **<0.001** | +0 | - | - | - | - |
| Serotype | Other† | Ref. | - | Ref. | - | - | Ref. | - | Ref. | - | - | Ref. | **<0.001** | Ref. | - | - | **157.50 (24)** | **<0.001** | **200.51 (24)** | **<0.001** |
|  | 1 | **0.38 (0.16–0.90)** | **0.028** | **0.25 (0.12–0.52)** | **0.001** | -52 | **0.16 (0.08–0.34)** | **<0.001** | **0.14 (0.07–0.27)** | **0.000** | -14 | **0.17 (0.08–0.36)** | <0.001 | **0.15 (0.07–0.30)** | **<0.001** | -13 | - | - | - | - |
|  | 3 | **2.24 (1.11–4.51)** | **0.024** | **1.81 (1.02–3.19)** | **0.042** | -24 | 1.32 (0.73–2.38) | 0.352 | 1.35 (0.80–2.28) | 0.261 | +2 | 1.05 (0.51–2.16**)** | 0.890 | 1.16 (0.60–2.25) | 0.642 | +9 | - | - | - | - |
|  | 4 | 1.00 (0.33–3.01) | 1.000 | 0.82 (0.33–2.07) | 0.680 | -22 | 0.41 (0.15–1.11) | 0.079 | **0.40 (0.17–0.94)** | **0.036** | -2 | **0.39 (0.15–1.04)** | 0.060 | **0.36 (0.15–0.89)** | **0.027** | -8 | - | - | - | - |
|  | 6A | **3.27 (1.33–8.07)** | **0.010** | **2.17 (0.97–4.84)** | **0.024** | -51 | **2.40 (1.06–5.43)** | **0.036** | 1.94 (0.90–4.19) | 0.092 | -24 | 1.70 (0.68–4.22) | 0.253 | 1.37 (0.57–3.28) | 0.469 | -24 | - | - | - | - |
|  | 6B | 2.21 (0.79–6.22) | 0.133 | 1.71 (0.70–4.20) | 0.238 | -29 | 2.00 (0.82–4.86) | 0.126 | 1.87 (0.83–4.22) | 0.133 | -7 | 1.56 (0.59–4.12) | 0.367 | 1.51 (0.61–3.73) | 0.364 | -3 | - | - | - | - |
|  | 6C | **3.00 (1.20–7.52)** | **0.019** | 1.63 (0.71–3.73) | 0.247 | -84 | **2.29 (1.09–5.99)** | **0.032** | 1.45 (0.67–3.16) | 0.347 | -58 | 1.22 (0.35–4.34) | 0.751 | 1.07 (0.30–3.71) | 0.913 | -14 | - | - | - | - |
|  | 7F | 0.77 (0.36–1.64) | 0.493 | **0.53 (0.28–0.99)** | **0.046** | -45 | **0.46 (0.24–0.86)** | **0.015** | **0.39 (0.22–0.68)** | **0.001** | -18 | 0.50 (0.24–1.03) | 0.060 | **0.39 (0.19–0.76)** | **0.007** | -28 | - | - | - | - |
|  | 8 | 0.76 (0.33–1.77) | 0.522 | **0.48 (0.23–0.98)** | **0.045** | -58 | 0.63 (0.32–1.16) | 0.185 | **0.46 (0.25–0.84)** | **0.011** | -37 | 0.72 (0.34–1.55) | 0.406 | 0.60 (0.29–1.22) | 0.163 | -20 | - | - | - | - |
|  | 9N | **4.15 (1.50**–**11.53)** | **0.006** | 2.40 (0.94–6.12) | 0.067 | -73 | 2.40 (0.93–6.21) | 0.071 | 1.63 (0.67–3.98) | 0.279 | -47 | 2.45 (0.79–7.60) | 0.119 | 1.60 (0.57–4.51) | 0.368 | -53 | - | - | - | - |
|  | 9V | 1.75 (0.63–4.83) | 0.280 | 1.38 (0.58–3.32) | 0.466 | -27 | 0.95 (0.40–2.28) | 0.913 | 0.85 (0.38–1.90) | 0.696 | -12 | 0.87 (0.34–2.19) | 0.762 | 0.78 (0.33–1.86) | 0.591 | -12 | - | - | - | - |
|  | 10A | 2.00 (0.56–7.09) | 0.283 | 1.33 (0.44–4.01) | 0.609 | -50 | 0.55 (0.14–2.11) | 0.380 | 0.38 (0.10–1.40) | 0.146 | -45 | **-** | - | - |  | - | - | - | - | - |
|  | 11A | **3.43 (1.22–9.67)** | **0.020** | **2.82 (1.20–6.67)** | **0.018** | -22 | **2.89 (1.11–7.54)** | **0.030** | **2.53 (1.08–5.92)** | **0.033** | -14 | 4.09 (1.00–16.72) | 0.050 | **4.28 (1.09–16.80)** | **0.037** | +4 | - | - | - | - |
|  | 12F | 0.91 (0.30–2.72) | 0.865 | 0.50 (0.18–1.40) | 0.186 | -82 | 0.85 (0.37–1.95) | 0.695 | 0.56 (0.26–1.23) | 0.151 | -52 | 0.47 (0.17–1.26) | 0.132 | **0.35 (0.13–0.92)** | **0.034** | -34 | - | - | - | - |
|  | 14 | 1.24 (0.46–3.33) | 0.676 | 1.00 (0.43–2.34) | 1.000 | -24 | 1.20 (0.55–2.62) | 0.647 | 1.13 (0.55–2.31) | 0.735 | -6 | 1.23 (0.52–2.87) | 0.636 | 1.29 (0.58–2.87) | 0.522 | 5 | - | - | - | - |
|  | 18C | 0.86 (0.23–3.26) | 0.821 | 0.50 (0.14–1.79) | 0.288 | -72 | 0.67 (0.24–1.86) | 0.439 | 0.47 (0.17–1.25) | 0.130 | -43 | 0.70 (0.25–1.97) | 0.502 | 0.64 (0.24–1.70) | 0.374 | -9 | - | - | - | - |
|  | 19A | 1.63 (0.80–3.34) | 0.182 | 1.05 (0.58–1.92) | 0.862 | -55 | 0.67 (0.36–1.24) | 0.198 | **0.53 (0.30–0.92)** | **0.025** | -26 | 0.74 (0.35–1.57) | 0.428 | 0.61 (0.30–1.25) | 0.183 | -21 | - | - | - | - |
|  | 19F | **3.60 (1.32–9.80)** | **0.012** | **2.67 (1.14–6.25)** | **0.024** | -35 | **2.80 (1.11–7.07)** | **0.029** | **2.45 (1.07–5.61)** | **0.034** | -14 | 1.84 (0.64–5.30) | 0.258 | 1.87 (0.68–5.14) | 0.222 | +2 | - | - | - | - |
|  | 20 | 1.38 (0.35–5.49) | 0.643 | 1.00 (0.31–3.25) | 1.000 | -38 | 0.67 (0.20–2.25) | 0.396 | 0.54 (0.18–1.61) | 0.272 | -24 | 1.02 (0.27–3.80) | 0.973 | 0.91 (0.27–3.06) | 0.890 | -12 | - | - | - | - |
|  | 22F | 1.40 (0.35–5.46) | 0.397 | 1.12 (0.59–2.10) | 0.732 | -25 | 0.74 (0.39–1.43) | 0.371 | 0.81 (0.46–1.42) | 0.453 | +9 | 0.96 (0.44–2.07) | 0.915 | 0.94 (0.46–1.92) | 0.877 | -2 | - | - | - | - |
|  | 23A | 2.00 (0.63–6.38) | 0.124 | 1.33 (0.48–3.71) | 0.581 | -50 | 2.22 (0.81–6.07) | 0.119 | 1.50 (0.61–3.71) | 0.382 | -48 | 1.43 (0.42–4.88) | 0.566 | 1.37 (0.45–4.19) | 0.573 | -4 | - | - | - | - |
|  | 23F | 1.50 (0.55–4.09) | 0.428 | 1.20 (0.51–2.86) | 0.679 | -25 | 1.04 (0.46–2.39) | 0.920 | 0.92 (0.43–1.96) | 0.819 | -13 | 1.47 (0.61–3.57) | 0.392 | 1.33 (0.58–3.08) | 0.493 | -11 | - | - | - | - |
|  | 31 | 2.00 (0.56–7.09) | 0.283 | 2.33 (0.83–6.54) | 0.107 | +14 | 0.73 (0.21–2.48) | 0.611 | 0.89 (0.31–2.59) | 0.833 | +18 | 1.64 (0.33–8.10 | 0.546 | 1.42 (0.29–6.95) | 0.659 | -15 | - | - | - | - |
|  | 33F | 2.16 (0.84–5.58) | 0.112 | 1.33 (0.56–3.19) | 0.518 | -62 | 1.20 (0.52–2.79) | 0.672 | 0.89 (0.40–1.99) | 0.779 | -35 | 1.47 (0.54–4.05) | 0.453 | 1.39 (0.52–3.68) | 0.504 | -6 | - | - | - | - |
|  | 35F | 1.38 (0.35–5.49) | 0.643 | 0.63 (0.17–2.30) | 0.486 | -119 | 1.71 (0.53–5.57) | 0.370 | 0.95 (0.35–2.62) | 0.926 | -80 | 1.47 (0.40–5.48) | 0.564 | 0.91 (0.27–3.06) | 0.890 | -62 | - | - | - | - |
| Clinical presentation | Other | Ref. | - | Ref. | - | - | Ref. | **-** | Ref. | - | - | Ref. | 0.007 | (-) |  |  | **27.32 (3)** | **<0.001** | **33.84 (3)** | **<0.001** |
|  | Meningitis | 0.81 (0.44–1.51) | 0.513 | 0.81 (0.46–1.45) | 0.487 | +0 | **0.44 (0.25–0.79)** | **0.006** | **0.43 (0.25–0.74)** | **0.002** | -2 | **0.41 (0.22–0.79)** | **0.007** | 0.37 (0.20-0.69) | 0.002 | -11 | - | - | - | - |
|  | Pneumonia | 0.88 (0.57–1.35) | 0.557 | 0.97 (0.66–1.44) | 0.896 | +9 | 0.80 (0.55–1.15) | 0.228 | 0.84 (0.60–1.18) | 0.315 | +5 | 1.04 (0.69–1.55) | 0.865 | 1.00 (0.68-1.46) | 0.988 | -4 | - | - | - | - |
|  | Septiceamia | **1.86 (1.07–3.23)** | **0.027** | **1.96 (1.19–3.24)** | **0.008** | +5 | 1.19 (0.72–1.98) | 0.497 | 1.29 (0.81–2.05) | 0.283 | +8 | 1.28 (0.69–2.37) | 0.434 | 1.17 (0.65-2.08) | 0.595 | -9 | - | - | - | - |
| Alcohol misuse | No | Ref. | - | Ref. | - | - | Ref. | - | Ref. | - |  | Ref. | - | Ref. | - | - | 0.01 (1) | 0.916 | 0.97 (1) | 0.325 |
|  | Yes | 1.01 (0.61–1.66) | - | 1.37 (0.90–2.07) | 0.139 | +26 | 0.76 (0.48–1.20) | 0.240 | 1.00 (0.65–1.50) | 0.991 | +24 | 0.91 (0.55–1.48) | 0.692 | 0.99 (0.61–1.59) | 0.975 | +8 | - | - | - | - |
| Chronic heart disease | No | Ref. | 0.973 | Ref. | - | - | Ref. | - | Ref. | - |  | Ref. | - | Ref. | - | - | **132.46 (1)** | **<0.001** | **150.59 (1)** | **<0.001** |
|  | Yes | **2.61 (1.93–3.53)** | - | **2.75 (2.07–3.65)** | **<0.001** | +5 | **3.23 (2.45–4.28)** | **<0.001** | **3.41 (2.59–4.49)** | **<0.001** | +5 | **4.11 (2.95–5.73)** | **<0.001** | **4.26 (3.04–5.97)** | **<0.001** | +4 | - | - | - | - |
| Chronic liver disease | No | Ref. | **<0.001** | Ref. | - | - | Ref. | - | Ref. | - |  | Ref. | - | Ref. | - | - | **16.59 (1)** | **<0.001** | **17.49 (1)** | **<0.001** |
|  | Yes | **1.89 (1.06–3.39)** | - | **1.99 (1.18–3.34)** | **0.010** | +5 | 1.63 (0.94–2.84) | 0.084 | **1.67 (0.98–2.81)** | **0.041** | +2 | 1.82 (0.93–3.55) | 0.079 | **1.87 (0.97–3.64)** | **0.043** | +3 | - | - | - | - |
| Chronic lung disease | No | Ref. | **0.032** | Ref. | - | - | Ref. | - | Ref. | - |  | Ref. | - | Ref. | - | - | **55.39 (1)** | **<0.001** | **64.16 (1)** | **<0.001** |
|  | Yes | **1.72 (1.27–2.32)** | - | **1.86 (1.40–2.47)** | **<0.001** | +8 | **1.89 (1.44–2.47)** | **<0.001** | **1.93 (1.48–2.52)** | **<0.001** | +2 | **2.79 (2.05–3.81)** | **<0.001** | **2.66 (1.95–3.63)** | **<0.001** | -5 | - | - | - | - |
| Chronic renal disease | No | Ref. | **<0.001** | Ref. | - | - | Ref. | - | Ref. | - |  | Ref. | - | Ref. | - | - | **23.77 (1)** | **<0.001** | **35.28 (1)** | **<0.001** |
|  | Yes | **1.90 (1.28–2.82)** | - | **1.91 (1.33–2.75)** | **0.001** | +1 | **1.84 (1.27–2.68)** | **0.001** | **1.98 (1.38–2.84)** | **<0.001** | +7 | **1.85 (1.20–2.84)** | **0.005** | **2.03 (1.32–3.12)** | **<0.001** | +9 | - | - | - | - |
| Diabetes | No | Ref. | **0.002** | Ref. | - | - | Ref. | - | Ref. | - |  | Ref. | - | Ref. | - | - | **4.52 (1)** | **0.033** | **4.43 (1)** | **0.035** |
|  | Yes | 1.45 (0.97–2.17) | - | **1.42 (0.98–2.06)** | **0.026** | -2 | **1.49 (1.04­–2.15)** | **0.031** | **1.48 (1.04–2.10)** | **0.023** | -1 | 1.53 (1.01–2.34) | 0.046 | **1.57 (1.03–2.39)** | **0.026** | +3 | - | - | - | - |
| Immunosuppression | No | Ref. | 0.072 | Ref. | - | - | Ref. | - | Ref. | - |  | Ref. | - | Ref. | - | - | **63.00 (1)** | **<0.001** | **63.85 (1)** | **<0.001** |
|  | Yes | **1.67 (1.12–2.27)** | - | **1.62 (1.11–2.36)** | **0.012** | -3 | **2.73 (2.06–4.23)** | **<0.001** | **273 (1.91–3.89)** | **<0.001** | +0 | **2.77 (1.77–4.34)** | **<0.001** | **2.40 (1.54–2)** | **<0.001** | -15 | - | - | - | - |
| Number of risk factors | 0 | Ref. | **0.012** | Ref. | - | - | Ref. | **-** | Ref. | - |  | Ref. | **<0.001** | Ref. | - | - | **193.09 (2)** | **<0.001** | **194.75 (2)** | **<0.001** |
|  | 1 | **2.89 (2.03–4.12)** | **<0.001** | **2.20 (1.63–2.98)** | **<0.001** | -31 | **2.78 (2.05–3.80)** | **<0.001** | **2.27 (1.73–2.99)** | **<0.001** | -22 | **3.41 (2.46–4.74)** | **<0.001** | **2.77 (2.05–3.75)** | **<0.001** | -23 | **-** | **-** | **-** | **-** |
|  | ≥2 | **3.63 (2.53–5.23)** | **<0.001** | **2.93 (2.13–4.03)** | **<0.001** | -24 | **4.73 (3.43–6.52)** | **<0.001** | **4.06 (3.04–5.43)** | **<0.001** | -17 | **5.82 (4.05–8.37)** | **<0.001** | **4.99 (3.54–7.02)** | **<0.001** | -17 | **-** | **-** | **-** | **-** |

## Supplementary Table S5. Sensitivity analysis for adjusted associations with mortality post-diagnosis with IPD.

| **Variable** | **Category** | **30-day survival** | | | | | **12-month survival** | | | | | **36-month survival** | | | | | **Longer term survival (<7 years)** | | | | |
| --- | --- | --- | --- | --- | --- | --- | --- | --- | --- | --- | --- | --- | --- | --- | --- | --- | --- | --- | --- | --- | --- |
|  |  | **Complete cases only (n=1316)** | | **Full dataset (n=1521)** | | **% Δ OR** | **Complete cases only (n=1233)** | | **Full dataset (n=1329)** | | **% Δ OR** | **Complete cases only (n=906)** | | **Full dataset (n=950)** | | **% Δ OR** | **Complete cases only (n=1269), main effects** | | **Full dataset (n=1381), main effects** | | **% Δ HR** |
|  |  | **OR_AD_ (95% CI)** | **P** | **OR_AD_ (95% CI)** | **P** |  | **OR_AD_ (95% CI)** | **P** | **OR_AD_ (95% CI)** | **P** |  | **OR_AD_ (95% CI)** | **P** | **OR_AD_ (95% CI)** | **P** |  | **HR (95% CI)** | **P** | **OR_AD_ (95% CI)** | **P** |  |
| Age group† | 0-19 | Ref. | - | Ref. | - | - | Ref. | - | Ref. | - | - | Ref. | - | Ref. | - | - | Ref. | **<0.001** | Ref. | - |  |
|  | 20–39 | 0.98 (0.36–2.67) | 0.966 | 0.92 (0.35–2.45) | 0.871 | -7 | **1.77 (0.72–4.37)** | **0.215** | **1.73 (0.70–4.26)** | **0.231** | -2 | 1.73 (0.59–5.07) | 0.317 | 1.46 (0.51–4.18) | 0.481 | -18 | 1.83 (0.87­–3.88) | 0.114 | 1.83 (0.87–3.88) | 0.113 | +0 |
|  | 40–59 | 2.04 (0.97–4.28) | 0.060 | **2.18 (1.09–4.36)** | **0.028** | +6 | **3.09 (1.50–6.35)** | **0.002** | **3.41 (1.68–6.90)** | **0.001** | +9 | **4.17 (1.81–9.62)** | **<0.001** | **3.91 (1.77–8.63)** | **0.001** | -7 | 1.93 (0.90–4.13) | 0.091 | 2.06 (0.98–4.36) | 0.058 | +6 |
|  | 60–79 | **2.96 (1.46–5.98)** | **0.003** | **3.46 (1.78–6.71)** | **<0.001** | +14 | **6.16 (3.11–12.20)** | **<0.001** | **6.99 (3.55–13.76)** | **<0.001** | +12 | **12.30 (5.55–27.26)** | **<0.001** | **11.31 (5.30–24.15)** | **<0.001** | -9 | **2.49 (1.23–5.06)** | **0.011** | **2.56 (1.26–5.18)** | **0.009** | +3 |
|  | ≥80 | **6.58 (3.20–13.50)** | **<0.001** | **7.05 (3.58–13.87)** | **<0.001** | +7 | **17.76 (8.71–36.19)** | **<0.001** | **18.86 (9.30–38.24)** | **<0.001** | +6 | **62.86 (26.54–148.90)** | **<0.001** | **57.93 (25.34–132.45)** | **<0.001** | -8 | **4.60 (2.25–9.39)** | **<0.001** | **4.69 (2.30–9.57)** | **<0.001** | +2 |
| Sex† | Female | Ref. | - | Ref. | - | - | Ref. | - | Ref. | - | - | Ref. | - | Ref. | - | - | Ref. | - | Ref. | - |  |
|  | Male | 1.28 (0.94–1.75) | 0.116 | **1.44 (1.08–1.91)** | **0.012** | +11 | **1.64 (1.23–2.45)** | **0.001** | **1.77 (1.33–2.40)** | **<0.001** | +7 | **1.96 (1.37–2.81)** | **<0.001** | **1.92 (1.36–2.73)** | **<0.001** | -2 | 1.18 (0.88–1.60) | 0.268 | **1.26 (0.94–1.69)** | **0.119** | +6 |
| Year of diagnosis | 2006–07 | - | - | - | - | - | - | - | - | - | - | - | - | - | - | - | - | - | - | - |  |
|  | 2007­–08 | - | - | - | - | - | - | - | - | - | - | - | - | - | - | - | - | - | - | - |  |
|  | 2008–09 | - | - | - | - | - | - | - | - | - | - | - | - | - | - | - | - | - | - | - |  |
|  | 2009–10 | - | - | - | - | - | - | - | - | - | - | - | - | - | - | - | - | - | - | - |  |
|  | 2010­–11 | - | - | - | - | - | - | - | - | - | - | - | - | - | - | - | - | - | - | - |  |
|  | 2011–12 | - | - | - | - | - | - | - | - | - | - | - | - | - | - | - | - | - | - | - |  |
|  | 2012–13 | - | - | - | - | - | - | - | - | - | - | - | - | - | - | - | - | - | - | - |  |
| Deprivation | Quintile 1 | Ref. | - | Ref. | - | - | - | - | - | - | - | Ref. | - | Ref. | - | - | Ref. | **0.006** | Ref. | - |  |
|  | Quintile 2 | 1.33 (0.75–2.37) | 0.336 | 1.10 (0.65–1.86) | 0.761 | -21 | - | - | - | - | -- | 0.85 (0.44–1.63) | 0.621 | 0.71 (0.38–1.34) | 0.293 | -20 | 1.01 (0.73–1.40) | 0.956 | 0.96 (0.69–1.32) | 0.781 | -5 |
|  | Quintile 3 | 1.50 (0.85–2.64) | 0.165 | 1.26 (0.76–2.10) | 0.374 | -19 | - | - | - | - | - | 1.60 (0.85–3.00) | 0.144 | 1.34 (0.73–2.45) | 0.343 | -19 | 1.27 (0.93–1.75) | 0.138 | 1.25 (0.92–1.70) | 0.159 | -2 |
|  | Quintile 4 | **2.30 (1.34–3.95)** | **0.003** | **1.96 (1.21–3.17)** | **0.007** | -17 | **-** | **-** | **-** | **-** | - | **2.18 (1.18–4.01)** | **0.012** | **1.87 (1.04–3.37)** | **0.036** | -17 | **1.60 (1.18–2.17)** | **0.003** | **1.57 (1.17–2.12)** | **0.003** | -2 |
|  | Quintile 5 | 1.50 (0.87–2.60) | 0.647 | 1.37 (0.84–2.22) | 0.206 | -9 | - | - | - | - | - | 1.47 (0.80–2.68) | 0.213 | 1.30 (0.73–2.32) | 0.373 | -13 | 1.27 (0.93–1.72) | 0.135 | 1.25 (0.93–1.69) | 0.146 | -2 |
| Immunisation | Vaccinated | - | - | - | - | - | - | - | - | - | - | - | - | - | - | - | - | - | - | - |  |
|  | Unvaccinated | - | - | - | - | - | - | - | - | - | - | **-** | **-** | **-** | **-** | - | - | - | - | - |  |
| Serotype | Other† | Ref. | - | Ref. | - | - | - | - | Ref. | - | - | Ref. | - | Ref. | - | - | Ref. | **0.002** | Ref. | - |  |
|  | 1 | 0.81 (0.33–1.99) | 0.643 | 0.62 (0.28–1.41) | 0.254 | -31 | **0.30 (0.13–0.66)** | **0.003** | **0.27 (0.13–0.57)** | **0.001** | -11 | **0.33 (0.13–0.80)** | **0.015** | **0.32 (0.14–0.77)** | **0.011** | -3 | **0.51 (0.30–0.86)** | **0.011** | **0.49 (0.29–0.82)** | **0.006** | -4 |
|  | 3 | **2.20 (1.05–4.60)** | **0.036** | **2.05 (1.09–3.88)** | **0.027** | -7 | 1.22 (0.64–2.34) | 0.549 | 1.34 (0.72–2.49) | 0.358 | +9 | 0.89 (0.37–2.18) | 0.802 | 1.04 (0.44–2.43) | 0.930 | +14 | 1.08 (0.73–1.61) | 0.700 | 1.11 (0.76–1.63) | 0.585 | +3 |
|  | 4 | 1.22 (0.38–3.94) | 0.742 | 1.06 (0.37–3.03) | 0.926 | -15 | 0.43 (0.15–1.26) | 0.124 | 0.39 (0.13–1.13) | 0.083 | -10 | 0.31 (0.09–1.01) | 0.051 | 0.31 (0.10–1.02) | 0.053 | +0 | 0.77 (0.40–1.48) | 0.432 | 0.76 (0.40–1.45) | 0.407 | -1 |
|  | 6A | **3.11 (1.19–8.17)** | **0.021** | **2.68 (1.11–6.49)** | **0.029** | -16 | 2.10 (0.84–5.25) | 0.113 | 1.76 (0.72–4.30) | 0.214 | -19 | 1.42 (0.47–4.31) | 0.533 | 1.26 (0.43–3.69 | 0.670 | -13 | 1.34 (0.77–2.30) | 0.297 | 1.34 (0.78–2.30) | 0.292 | +0 |
|  | 6B | 2.12 (0.71–6.30) | 0.177 | 1.80 (0.67–4.85) | 0.244 | -18 | 1.63 (0.62–4.29) | 0.327 | 1.44 (0.55–3.77) | 0.455 | -13 | 1.16 (0.37–3.60) | 0.796 | 1.16 (0.38–3.56) | 0.790 | +0 | 1.33 (0.74–2.38) | 0.346 | 1.32 (0.74–2.37) | 0.344 | -1 |
|  | 6C | 2.33 (0.88–6.20) | 0.089 | 1.53 (0.62–3.74) | 0.357 | -52 | 1.70 (0.68–4.22) | 0.249 | 1.53 (0.63–3.76) | 0.350 | -11 | 0.89 (0.20–4.01) | 0.884 | 0.89 (0.20–3.97) | 0.879 | +0 | 1.21 (0.70–2.09) | 0.487 | 1.21 (0.70–2.07) | 0.492 | +0 |
|  | 7F | 1.12 (0.51–2.49) | 0.776 | 0.92 (0.46–1.83) | 0.806 | -22 | 0.62 (0.31–1.23) | 0.168 | 0.62 (0.32–1.19) | 0.149 | +0 | 0.66 (0.27–1.58) | 0.347 | 0.67 (0.29–1.57) | 0.357 | +1 | 0.68 (0.43–1.09) | 0.107 | 0.71 (0.46–1.11) | 0.137 | +4 |
|  | 8 | 0.83 (0.35–2.00) | 0.680 | 0.61 (0.28–1.35) | 0.225 | -36 | 0.54 (0.26–1.12) | 0.096 | **0.46 (0.23–0.94)** | **0.032** | -17 | 0.47 (0.19–1.17) | 0.104 | 0.52 (0.21–1.26) | 0.148 | +10 | 0.81 (0.52–1.27) | 0.357 | 0.80 (0.51–1.24) | 0.311 | -1 |
|  | 9N | **4.48 (1.50–13.37)** | **0.007** | **3.15 (1.14–8.69)** | **0.027** | -42 | 2.35 (0.82–6.70) | 0.110 | 2.13 (0.75–6.04) | 0.155 | -10 | 2.56 (0.65–10.20) | 0.181 | 2.62 (0.66–10.38) | 0.169 | +2 | **2.37 (1.29–4.37)** | **0.007** | **2.37 (1.29–4.36)** | **0.005** | +0 |
|  | 9V | 1.50 (0.52–4.35) | 0.453 | 1.51 (0.59–3.87) | 0.391 | +1 | 0.63 (0.24–1.64) | 0.348 | 0.66 (0.26–1.66) | 0.379 | +5 | 0.39 (0.13–1.16) | 0.089 | 0.44 (0.15–1.27) | 0.129 | +11 | 0.95 (0.55–1.65) | 0.864 | 0.98 (0.57–1.67) | 0.939 | +3 |
|  | 10A | 2.63 (0.66–10.42) | 0.169 | 1.81 (0.54–6.09) | 0.341 | -45 | 0.61 (0.13–2.81) | 0.529 | 0.56 (0.12–2.54) | 0.453 | -9 | - | - | - | - | - | 0.96 (0.34–2.71) | 0.942 | 1.05 (0.41–2.68) | 0.913 | +9 |
|  | 11A | 2.66 (0.90–7.91) | 0.078 | **2.67 (1.04–6.85)** | **0.042** | +0 | 2.39 (0.84–6.80) | 0.103 | 2.55 (0.92–7.06) | 0.070 | +6 | 2.76 (0.55–13.89) | 0.219 | 3.58 (0.74–17.41) | 0.114 | +23 | 1.68 (0.96–2.93) | 0.067 | **1.78 (1.03–3.06)** | **0.038** | +6 |
|  | 12F | 0.93 (0.30–2.91) | 0.900 | 0.67 (0.23–1.97) | 0.465 | -39 | 0.80 (0.32–1.98) | 0.625 | 0.62 (0.26–1.50) | 0.289 | -29 | 0.42 (0.13–1.32) | 0.138 | 0.42 (0.13–1.30) | 0.133 | +0 | 0.73 (0.39–1.35) | 0.311 | 0.66 (0.36–1.24) | 0.198 | -11 |
|  | 14 | 1.12 (0.40–3.18) | 0.825 | 1.06 (0.42–2.63) | 0.906 | -6 | 0.67 (0.27–1.62) | 0.370 | 0.59 (0.25–1.41) | 0.239 | -14 | 0.64 (0.22–1.90) | 0.427 | 0.71 (0.25–2.05) | 0.532 | +10 | 0.92 (0.55–1.55) | 0.765 | 0.94 (0.56–1.56) | 0.807 | +2 |
|  | 18C | 0.79 (0.19–3.22) | 0.738 | 0.63 (0.16–2.48) | 0.513 | -25 | 0.61 (0.20–1.90) | 0.399 | 0.57 (0.19–1.76) | 0.330 | -7 | 0.68 (0.19–2.44) | 0.555 | 0.92 (0.26–3.18) | 0.891 | +26 | 1.25 (0.62–2.50) | 0.530 | 1.26 (0.63–2.51) | 0.515 | +1 |
|  | 19A | 1.55 (0.72–3.32) | 0.259 | 1.21 (0.62–2.36) | 0.578 | -28 | 0.52 (0.26–1.02) | 0.058 | **0.44 (0.23–0.85)** | **0.015** | -18 | 0.48 (0.19–1.24) | 0.129 | 0.46 (0.18–1.16) | 0.099 | -4 | 0.80 (0.53–1.22) | 0.305 | 0.76 (0.50–1.15) | 0.196 | -5 |
|  | 19F | **3.90 (1.32–11.49)** | **0.013** | **3.44 (1.35–8.77)** | **0.010** | -13 | **3.27 (1.13–9.43)** | **0.029** | 3.11 (1.09–8.83) | 0.033 | -5 | 2.27 (0.64–8.51) | 0.225 | 2.23 (0.60–8.25) | 0.230 | -2 | 1.64 (0.91–2.97) | 0.100 | 1.70 (0.95–3.02) | 0.072 | +4 |
|  | 20 | 1.20 (0.28–5.07) | 0.804 | 0.89 (0.23–3.59) | 0.880 | -35 | 0.51 (0.14–1.84) | 0.300 | 0.56 (0.17–1.83) | 0.336 | +9 | 0.63 (0.14–2.80) | 0.544 | 0.63 (0.14–2.78) | 0.540 | +0 | 0.70 (0.31–1.57) | 0.390 | 0.70 (0.31–1.56) | 0.381 | +0 |
|  | 22F | 1.26 (0.56–2.83) | 0.575 | 0.98 (0.48–1.98) | 0.946 | -29 | 0.56 (0.27–1.16) | 0.120 | 0.58 (0.30–1.15) | 0.119 | +3 | 0.86 (0.34–2.19) | 0.749 | 0.79 (0.32–1.94) | 0.601 | -9 | 0.83 (0.54–1.27) | 0.387 | 0.83 (0.54–1.26) | 0.375 | +0 |
|  | 23A | 1.34 (0.40–4.55) | 0.635 | 1.38 (0.46–4.17) | 0.565 | +3 | 1.49 (0.49–4.52) | 0.479 | 1.02 (0.37–2.33) | 0.975 | -46 | 0.85 (0.19–3.78) | 0.829 | 0.84 (0.19–3.68) | 0.816 | -1 | 1.33 (0.72–2.44) | 0.358 | 1.34 (0.73–2.46) | 0.323 | +1 |
|  | 23F | 1.42 (0.49–4.08) | 0.517 | 1.28 (0.49–3.34) | 0.620 | -11 | 0.69 (0.28–1.72) | 0.424 | 0.68 (0.28–1.66) | 0.399 | -1 | 0.75 (0.25–2.19) | 0.592 | 0.79 (0.27–2.26) | 0.655 | +5 | 0.70 (0.41–1.21) | 0.202 | 0.72 (0.42–1.22) | 0.223 | +3 |
|  | 31 | 1.59 (0.41–6.14) | 0.499 | 2.01 (0.65–6.22) | 0.228 | +21 | 0.40 (0.11–1.50) | 0.176 | 0.37 (0.10–1.38) | 0.141 | -8 | 0.46 (0.08–2.67) | 0.391 | 0.46 (0.08–2.61) | 0.379 | +0 | 0.96 (0.48–1.93) | 0.908 | 0.94 (0.47–1.89) | 0.870 | -2 |
|  | 33F | 2.33 (0.84–6.49) | 0.104 | 1.73 (0.66–4.48) | 0.262 | -35 | 1.08 (0.42–2.79) | 0.876 | 0.92 (0.36–2.33) | 0.858 | -17 | 1.53 (0.43–5.45) | 0.515 | 1.54 (0.44–5.42) | 0.498 | +1 | 1.31 (0.76–2.26) | 0.328 | 1.26 (0.73–2.16) | 0.404 | -4 |
|  | 35F | 0.86 (0.20–3.64) | 0.839 | 0.57 (0.15–2.21) | 0.413 | -51 | 1.35 (0.36–5.04) | 0.653 | 1.13 (0.32–4.02) | 0.845 | -19 | 1.58 (0.30–8.34) | 0.590 | 1.54 (0.30–7/94) | 0.605 | -3 | 0.89 (0.42–1.90) | 0.763 | 0.87 (0.41–1.85) | 0.717 | -2 |
| Clinical presentation | Other | Ref. | - | Ref. | - | - | - | - | - | - | - | - | - | - | - | - | Ref. | **0.039** | Ref. | - |  |
|  | Meningitis | 1.33 (0.67–2.67) | 0.416 | 1.26 (0.66–2.41) | 0.478 | -6 | - | - | - | - | - | - | - | - | - | - | 1.25 (0.69–2.26) | 0.469 | 1.19 (0.65–2.16) | 0.571 | -5 |
|  | Pneumonia | 0.86 (0.53–1.40) | 0.557 | 0.93 (0.60–1.43) | 0.733 | +8 | - | - | - | - | - | - | - | - | - | - | 0.77 (0.59–1.00) | 0.052 | 0.78 (0.60–1.01) | 0.057 | +1 |
|  | Septicaemia | 1.70 (0.92–3.14) | 0.091 | 1.73 (1.00–3.01) | 0.051 | +2 | - | - | - | - | - | - | - | - | - | - | 1.02 (0.72­–1.45) | 0.925 | 0.99 (0.70–1.40) | 0.972 | -3 |
| Alcohol misuse | No | - | - | - | - | - | - | - | - | - | - | - | - | - | - | - | - | - | - | - |  |
|  | Yes | - | - | - | - | - | - | - | - | - | - | - | - | - | - | - | - | - | - | - |  |
| Chronic heart disease | No | - | - | - | - | - | - | - | - | - | - | Ref. | - | Ref. | - | - | Ref. | - | Ref. | - |  |
|  | Yes | - | - | - | - | - | - | - | - | - | - | **1.69 (1.11–2.57)** | **0.014** | **1.76 (1.16–2.66)** | **0.008** | +4 | **1.33 (1.10–1.61)** | **0.004** | **1.34 (1.12–1.62)** | **0.002** | +1 |
| Chronic liver disease | No | - | - | - | - | - | - | - | - | - | - | Ref. | - | Ref. | - | - | Ref. | - | Ref. | - |  |
|  | Yes | - | - | - | - | - | - | - | - | - | - | **3.34 (1.47–7.63)** | **0.004** | **3.58 (1.62–7.91)** | **0.002** | +7 | **2.30 (1.62–3.27)** | **<0.001** | **2.35 (1.67–3.30)** | **<0.001** | +2 |
| Chronic lung disease | No | - | - | - | - | - | - | - | - | - | - | Ref. | - | Ref. | - | - | Ref. | - | Ref. | - |  |
|  | Yes | - | - | - | - | - | - | - | - | - | - | **2.08 (1.39–3.12)** | **<0.001** | **1.99 (1.33–2.96)** | **0.001** | -5 | **1.29 (1.05–1.96)** | **0.008** | **1.29 (1.07–1.56)** | **0.007** | +0 |
| Chronic renal disease | No | - | - | - | - | - | Ref. | - | Ref. | - | - | Ref. | - | Ref. | - | - | - | - | - | - |  |
|  | Yes | - | - | - | - | - | **0.61 (0.38–0.99)** | **0.048** | 0.68 (0.43–1.07) | 0.092 | +10 | **0.55 (0.31–0.96)** | **0.036** | 0.60 (0.35**–1.03)** | 0.063 | +8 | - | - | - | - |  |
| Diabetes | No | - | - | - | - | - | - | - | - | - | - | - | - | - | - | - | - | - | - | - |  |
|  | Yes | - | - | - | - | - | - | - | - | - | - | - | - | - | - | - | - | - | - | - |  |
| Immunosuppression | No | - | - | - | - | - | Ref. | - | Ref. | - | - | Ref. | - | Ref. | - | - | Ref. | - | Ref. | - |  |
|  | Yes | - | - | - | - | - | **2.44 (1.57–3.81)** | **<0.001** | **2.28 (1.48–3.51)** | **<0.001** | -7 | **3.53 (2.01–6.19)** | **<0.001** | **3.42 (1.97–5.96)** | **<0.001** | -3 | 1.29 (0.85–1.96) | 0.229 | 1.27 (0.84–1.90) | 0.253 | -2 |
| Number of risk factors | 0 | Ref. | - | Ref. | - | - | Ref. | - | Ref. | - | - | - | - | - | - | - | - | - | - | - |  |
|  | 1 | **2.11 (1.43–3.13)** | **<0.001** | **1.81 (1.27–2.57)** | **0.001** | -17 | **1.64 (1.14–2.36)** | **0.008** | **1.54 (1.08–2.18)** | **0.016** | -6 | **-** | **-** | **-** | **-** | - | - | - | **-** | **-** |  |
|  | ≥2 | **2.17 (1.43–3.31)** | **0.019** | **1.95 (1.34–2.84)** | **0.001** | -11 | **2.22 (1.43–3.43)** | **<0.001** | **2.16 (1.42–3.28)** | **<0.001** | -3 | **-** | **-** | **-** | **-** | - | - | - | **-** | **-** |  |

Blue shading indicates significant (P<0.05) associations between individual serotypes and outcome.

## Supplementary Table S6. Model building for the Cox proportional hazards model for survival following IPD diagnosis, all cases.

| Model | Vs. | lnL | lnL statistic (P) | Variables (P) | Removed |
| --- | --- | --- | --- | --- | --- |
| M1 | - | -3418.745 | - | Age group (<0.001), sex (<0.001), deprivation (0.004), vaccination (0.070), serotype (<0.001), clinical presentation (0.029), chronic heart disease (0.171), chronic liver disease (<0.001), chronic lung disease (0.321), chronic renal disease (0.112), **diabetes (0.510)**, immunosuppression (0.002), number of risk factors (0.113) | - |
| M2 | M1 | -3418.966 | 0.44 (0.507) | Age group (<0.001), sex (<0.001), deprivation (0.005), vaccination (0.072), serotype (<0.001), clinical presentation (0.030), chronic heart disease (0.074), chronic liver disease (<0.001), chronic lung disease (0.136), **chronic renal disease (0.144)**, immunosuppression (<0.001), number of risk factors (0.123) | Diabetes |
| M3 | M2 | -3420.053 | 2.17 (0.140) | Age group (<0.001), sex (<0.001), deprivation (0.005), **vaccination (0.079)**, serotype (0.001), clinical presentation (0.033), chronic heart disease (0.015), chronic liver disease (<0.001), chronic lung disease (0.037), immunosuppression (<0.001), number of risk factors (0.074) | Chronic renal disease |
| M4 | M3 | -3421.61 | 3.11(0.078) | Age group (<0.001), sex (<0.001), deprivation (0.008), serotype (0.002), clinical presentation (0.043), chronic heart disease (0.010), chronic liver disease (<0.001), chronic lung disease (0.027), immunosuppression (<0.001), number of risk factors (0.061) | Vaccination |
| M5 | M4 | -3424.359 | 5.50 (0.064) | Age group (<0.001), sex (<0.001), deprivation (0.008), serotype (0.002), clinical presentation (0.024), chronic heart disease (0.002), chronic liver disease (<0.001), chronic lung disease (0.012), immunosuppression (<0.001) | Number of risk factors |
| M6 | M5 | -3395.136 | 58.45 (<0.001) | Age group (<0.001), sex (0.285), deprivation (0.005), serotype (0.001), clinical presentation (0.039), chronic heart disease (0.003), chronic liver disease (<0.001), chronic lung disease (0.008), immunosuppression (0.226); Time-dependent: Age group 3 (0.008), age group 4 (<0.001), age group 5 (<0.001), sex (0.026), **serotype 3 (0.119)**, meningitis (0.001), immunosuppression (0.021) |  |
| M7 | M6 | -3396.363 | 2.46 (0.117) | Age group (<0.001), sex (0.268), deprivation (0.006), serotype (0.002), clinical presentation (0.004), chronic heart disease (0.004), chronic liver disease (<0.001), chronic lung disease (0.008), immunosuppression (0.229); Time-dependent: Age group 3 (0.008), age group 4 (<0.001), age group 5 (<0.001), sex (0.030), meningitis (0.002) immunosuppression (0.001) | Serotype time-dependent |

lnL: log likelihood.

## Supplementary Table S7. Model building for the Cox proportional hazards model using an observation period starting 30 days after IPD diagnosis.

| Model | Vs. | lnL | lnL statistic (P) | Variables (P) | Removed |
| --- | --- | --- | --- | --- | --- |
| M1 | - | -1744.063 | - | Age group (<0.001), sex (<0.001), vaccination (0.269), serotype (0.023), clinical presentation (0.002), chronic heart disease (0.052), chronic liver disease (<0.001), chronic lung disease (0.040), chronic renal disease (0.164), immunosuppression (<0.001), **number of risk factors (0.751)** |  |
| M2 | M1 | -1744.349 | 0.57 (0.903) | Age group (<0.001), sex (<0.001), **vaccination (0.257)**, serotype (0.017), clinical presentation (0.002), chronic heart disease (0.018), chronic liver disease (<0.001), chronic lung disease (0.017), chronic renal disease (0.059), immunosuppression (<0.001) | Number of risk factors |
| M3 | M2 | -1744.995 | 1.29 (0.256) | Age group (<0.001), sex (<0.001), serotype (0.018), clinical presentation (0.002), chronic heart disease (0.011), chronic liver disease (<0.001), chronic lung disease (0.011), **chronic renal disease (0.062)**, immunosuppression (<0.001) | Vaccination |
| M4 | M3 | -1746.271 | 3.71 (0.054) | Age group (<0.001), sex (<0.001), serotype (0.027), clinical presentation (0.002), chronic heart disease (0.015), chronic liver disease (<0.001), chronic lung disease (0.014), immunosuppression (<0.001) | Chronic renal disease |
| M5 | M4 | -1737.788 | 16.97 (<0.001) | Age group (<0.001), sex (<0.001), serotype (<0.001), clinical presentation (0.002), chronic heart disease (0.019), chronic liver disease (0.686), chronic lung disease (0.016), immunosuppression (<0.001); Time-dependent: serotype 1 (0.201), serotype 35F (0.001), chronic liver disease (0.115) |  |
| M6 | M5 | -1738.827 | 1.87 (0.1713) | Age group (<0.001), sex (<0.001), serotype (<0.001), clinical presentation (0.002), chronic heart disease (0.017), chronic liver disease (0.711), chronic lung disease (0.016), immunosuppression (<0.001); Time-dependent: serotype 35F (0.001), chronic liver disease (0.122) | Serotype 1 time-dependent |
| M7 | M6 | -1740.193 | 2.73 (0.0984) | Age group (<0.001), sex (<0.001), serotype (<0.001), clinical presentation (0.002), chronic heart disease (0.019), chronic liver disease (<0.001), chronic lung disease (0.015), immunosuppression (<0.001); Time-dependent: serotype 35F (0.001) | Chronic liver disease time-dependent |

lnL: log likelihood.
